# Transforming evidence synthesis: A systematic review of the evolution of automated meta-analysis in the age of AI

**DOI:** 10.1017/rsm.2025.10065

**Published:** 2026-01-09

**Authors:** Lingbo Li, Anuradha Mathrani, Teo Susnjak

**Affiliations:** School of Mathematical and Computational Sciences, Massey University, New Zealand

**Keywords:** AI-driven meta-analysis, automated evidence synthesis, automated meta-analysis (AMA), large language models for meta-analysis, scalable meta-analysis, systematic reviews

## Abstract

Exponential growth in scientific literature has heightened the demand for efficient evidence-based synthesis, driving the rise of the field of automated meta-analysis (AMA) powered by natural language processing and machine learning. This PRISMA systematic review introduces a structured framework for assessing the current state of AMA, based on screening 13,216 papers (2006–2024) and analyzing 61 studies across diverse domains. Findings reveal a predominant focus on automating data processing (52.5%), such as extraction and statistical modeling, while only 16.4% address advanced synthesis stages. Just one study (approximately 2%) explored preliminary full-process automation, highlighting a critical gap that limits AMA’s capacity for comprehensive synthesis. Despite recent breakthroughs in large language models and advanced AI, their integration into statistical modeling and higher-order synthesis, such as heterogeneity assessment and bias evaluation, remains underdeveloped. This has constrained AMA’s potential for fully autonomous meta-analysis (MA). From our dataset spanning medical (67.2%) and non-medical (32.8%) applications, we found that AMA has exhibited distinct implementation patterns and varying degrees of effectiveness in actually improving efficiency, scalability, and reproducibility. While automation has enhanced specific meta-analytic tasks, achieving seamless, end-to-end automation remains an open challenge. As AI systems advance in reasoning and contextual understanding, addressing these gaps is now imperative. Future efforts must focus on bridging automation across all MA stages, refining interpretability, and ensuring methodological robustness to fully realize AMA’s potential for scalable, domain-agnostic synthesis.

## Highlights

### What is already known?

- Traditional meta-analysis (MA) is resource-intensive, struggles with scalability, and suffers from reproducibility issues, limiting its efficiency and reliability.
- Automated MA (AMA) has advanced with machine learning and specialized tools, improving data extraction, statistical synthesis, and expanding beyond medical research.
- Integration challenges remain, including workflow fragmentation, analytical limitations, and interoperability barriers, hindering full automation.

### What is new?

- This study presents a timely systematic and comparative analysis of AMA research progress and applications across medical and non-medical domains to reveal distinct patterns in implementation challenges and opportunities.
- This study introduces a structured analytical framework to systematically evaluate the alignment between technological solutions and specific meta-analytical tasks, ensuring more effective automation implementation.
- This study identifies gaps in current AMA capabilities and presents a roadmap for advancement, taking recent progress in AI, and specifically breakthroughs in large language models, into account.

### Potential impact for RSM readers

- For researchers, it maps AI-enhanced AMA tools, boosting efficiency in MA and inspiring cross-disciplinary innovation through AI’s transformative power.
- For tool developers, it highlights gaps in AI-driven heterogeneity and bias assessment, urging advanced AI integration for scalable, interdisciplinary tools.
- For policymakers, it emphasizes the vitality of AI-powered AMA for evidence-based decisions and the need for standardization and investment in comprehensive systems.

## 1 Introduction

Automation has become integral to modern life; it is driving efficiencies across industries and is now transforming knowledge-intensive domains such as academia.^1^ While businesses have long leveraged automation for operational gains,^2^ scholarly research is now accelerating the adoption of AI-driven tools to enhance both efficiency and scalability in evidence synthesis.^3^ Systematic literature reviews (SLRs) are a cornerstone of academic research and are also among the most resource-intensive academic endeavors, whose workflows stand to be revolutionized by recent advancements in natural language processing (NLP, a field of AI focused on enabling computers to understand and generate human language),^4^ machine learning (ML, algorithms that learn patterns from data to make predictions or decisions),^5^ ^,^ ^6^ and large language models (LLMs, a type of ML model trained on massive text corpora to perform advanced language tasks).^7^ These technologies are accelerating automation in literature curation, data extraction, and synthesis, and thereby addressing the growing challenge of processing vast and rapidly expanding volume of scientific outputs.^8^

Meta-analyses (MAs) represent a key methodology within the context of SLRs for aggregating quantitative findings^9^ ^,^ ^10^ for which the current technological advancements present both opportunities and challenges for further automation.^8^ Conducting MAs is resource-intensive, often spanning months or years. With the explosion in the number of papers being published in academic databases, researchers have estimated that the average time to complete and publish a systematic review requiring five researchers is 67 weeks, with an approximate cost of US$140,000.^11^ Moreover, robust MA reviews tend to require an engagement with 3–5 domain experts to ensure its thoroughness, reliability, and accuracy.^12^ Such heavy demands on time, human resource, and financial investment pose barriers toward getting timely evidence-based synthesis, particularly in disciplines where rapid and accurate decision-making is essential. Consequently, automation has gained traction across various MA stages to mitigate these constraints. Studies have applied AI-driven techniques to enhance efficiency in literature screening,^13^ ^–^ ^15^ data extraction,^16^ ^–^ ^19^ risk-of-bias assessment,^20^ and heterogeneity reduction.^21^ Despite these gains, automation efforts remain fragmented, with uneven progress across stages, particularly in those requiring complex reasoning and synthesis tasks.

While these advancements contributed toward progress in streamlining various stages of MAs in isolation, no comprehensive undertaking has been made recently to assess the current state of research on the automation of MAs and to situate the existing gaps within the significant and evolving breakthroughs in AI and LLMs, which are increasingly capable of performing complex reasoning.^22^ The only dedicated review^23^ synthesizing automated MA (AMA) focused narrowly on clinical trials and identified 38 approaches across 39 articles that applied ML techniques to various stages of MA. However, it concluded that automation remains “far from significantly supporting and facilitating the work of researchers.^23^” Clinical trials generally involve standardized procedures and well-defined outcomes that enable automation, while other domains, such as education and social sciences, exhibit more heterogeneous study designs and data formats, making automation more complex. While informative, this clinical trial-focused review has limited relevance for broader AMA research, as it overlooks recent methodological developments and predates advances in LLMs that could transform automation opportunities. Aside from this work, semi-AMA (SAMA) has also emerged as an interim solution, shortening MA timelines while maintaining rigor through expert.^24^ However, SAMA depends on human intervention in key steps, such as study selection and results interpretation, which limits its scalability. Given these shortcomings, a comprehensive review of AMA progress across domains is urgently needed to harness AI’s full potential and address persistent limitations in evidence synthesis automation.

Therefore, this study critically examines the current state of automation in MA research, identifying existing approaches and challenges in preparation for the next wave of AI-driven breakthroughs that are poised further transform the field. It addresses a critical gap by providing the first comprehensive and systematic synthesis of AMA applications across both medical and non-medical domains using a structured analytical framework. Through this analysis, we highlight key challenges and opportunities in AMA and offer insights into its evolving role in quantitative evidence synthesis. Our study therefore makes three meaningful contributions to AMA:

- First, it presents a timely systematic and comparative analysis of AMA research progress and applications across medical and non-medical domains to reveal distinct patterns in implementation challenges and opportunities.
- Second, it introduces a structured analytical framework to systematically evaluate the alignment between technological solutions and specific meta-analytical tasks, ensuring more effective automation implementation.
- Third, it identifies critical gaps in current AMA capabilities—such as the need for deeper analytical integration and enhanced evidence synthesis—and presents a roadmap for advancement taking the recent AI, and specifically LLM breakthroughs into account.

## 2 Background

The following section explores the history and evolution of MA, tracing its development from a relatively nascent statistical technique to its current prominence in evidence synthesis across disciplines. The second section discusses our analytical framework that informs on technology adoption and task characteristics for conducting the AMA research process. These have helped lay out the research questions for this study.

### 2.1 History and evolution of meta-analysis

MA originated from the pioneering work by Glass^25^ in the late 1970s, who developed a statistical framework for synthesizing research findings across educational and psychological studies, formally coining the term “MA.”^25^ The methodology expanded significantly into medicine and other scientific domains during the 1980s–1990s, particularly for analyzing randomized controlled trials (RCTs). This expansion was driven by the growing demand for evidence-based decision-making, enabling researchers to address contradictory results and overcome limitations of small sample sizes.^26^ One landmark application in cardiovascular medicine evaluated statin use in reducing cholesterol levels by pooling data from numerous clinical trials to demonstrate clear benefits in lowering heart disease risks, which ultimately provided compelling evidence.^27^ The field advanced further through more sophisticated statistical models and refined effect size estimation techniques,^28^ enhancing the robustness of quantitative synthesis. The development of Preferred Reporting Items for Systematic Reviews and Meta-Analyses (PRISMA) guidelines,^29^ ^–^ ^32^ with its most recent 2020 update, established rigorous reporting standards that minimize bias and improve finding reliability. MA has become instrumental in healthcare research, and as the highest level of evidence synthesis,^33^ MA provides critical insights for clinical guidelines and public health policies.

However, the exponential growth in published research—exemplified by ScienceDirect (https://www.sciencedirect.com/) with 16 million papers from 2,500 journals serving 25 million monthly researchers—has challenged traditional MA approaches. The growing volume of literature has necessitated the development of automation tools to streamline and expedite the review process. Scholars anticipate that automated systematic reviews will revolutionize evidence-based medicine through real-time analysis capabilities and optimized workflows.^5^ ^,^ ^8^ While various software packages (RevMan,^1^ Comprehensive Meta-Analysis,^2^ Stata,^3^ and SPSS^4^) support MA through features like effect size calculation and heterogeneity assessment, they are better characterized as “computer-assisted” rather than truly automated. For instance, RevMan, despite its user-friendly interface for MA, still requires substantial manual data extraction. Similarly, while Comprehensive Meta-Analysis offers advanced statistical modeling, and Stata and SPSS provide flexible analysis capabilities, they all demand significant user intervention and statistical expertise. In addition, the commercial nature and high licensing costs of them limit accessibility for researchers with limited funding.

Recent advancements in AI-driven techniques, including NLP, ML, and LLMs, have markedly improved the efficiency of MA. Automated processes now condense tasks—once requiring months and multiple authors—into days, leveraging enhanced computational methods. For instance, LLMs have demonstrated sensitivity approaching human performance in the initial screening of systematic reviews,^34^ illustrating their potential to streamline early AMA stages. Despite these improvements, full deployment of AMA remains in development, with current efforts limited in scope. Research has primarily focused on clinical trials^23^ and SAMA,^24^ where SAMA reducing timelines while relying on human oversight for rigor. This narrow emphasis restricts AMA’s broader deployment across diverse domains, highlighting an ongoing challenge in achieving comprehensive automation. Emerging AI “thinking models,”^35^ ^–^ ^37^ capable of performing complex reasoning, offer a promising avenue to bridge this gap. These models could automate sophisticated synthesis tasks, such as heterogeneity assessment and statistical integration, thereby enhancing AMA’s precision and scalability. For instance, their ability to adapt reasoning to varied research contexts holds potential for wider application, which points to a critical opportunity to advance evidence synthesis automation.^22^

### 2.2 Analytical frameworks for technology evaluation

Having a robust analytical framework is crucial to synthesizing data drawn from pertinent studies and in drawing meaningful conclusions. The choice of framework for technology evaluation depends on the relevancy of data collected and the research questions that have been posed to analyse this data for understanding aspects related to technology’s usage and its overall performance. Two analytical frameworks that can provide us with a comprehensive assessment on the usage of various information systems (ISs)/information technology (IT) systems are unified theory of acceptance and use of technology (UTAUT), proposed by Venkatesh et al.^38^ ^,^ ^39^ (for explaining user attitudes, their behavioral intentions, and overall acceptance of the technology in use) and task–technology fit (TTF), proposed by Goodhue and Thompson^40^ (for interpreting technology alignment with the proposed tasks that can lead to high-performance impacts). However, a key element missing in UTAUT is the disposition of the users, namely, users’ computer self-efficacy or their innovativeness in making best use of the technology; therefore, user expectancy (performance expectancy and effort expectancy) and contextual factors (facilitating conditions and social influences) have been proposed as extensions to UTAUT.^41^ TTF, on the other hand, posits that the effectiveness of technology adoption and its usage depends on how well the technology supports the specific needs of a given task. It emphasizes on characteristics of both the task and the technology to make a statement on task–technology fitness. If there is a good fit between task and technology, it increases the likelihood of technology utilization and leads to increased performance impact.

TTF explains technology adoption (e.g., data locatability, data quality, data accessibility, timeliness, technology reliability, and ease of use) by focusing on the actual usage of the technology which can in turn offer valuable insights on technology design and improvement strategies. We can delve deeper into the functional match between technology and tasks, which is particularly relevant for complex, multistage process like AMA, where task demands vary significantly at different stages. It lays a strong foundation to understand how technology characteristics would influence task behaviors and consequently the utilization of that technology for the given purpose, and finally to provide a measure of the performance impacts. These impacts could lead to further development of more tools and services already in the marketplace or lead to redesigning of tasks to take better advantage of the technology or to further embark on training programs to better engage users in using the technologies.^40^

In this study, TTF is applied at the task level, focusing on concrete AMA tasks at each phase rather than abstract task attributes.^42^ ^,^ ^43^ By applying TTF to AMA, we can evaluate the suitability of automation tools across various stages of AMA and assess how well available technologies support the specific tasks and user needs at each stage.

- *Task demands and technology support across AMA stages:* The distinct stages of AMA involve different types of tasks with varying demands and levels of complexity. For instance, in the data extraction phase, automation tools must handle unstructured data from diverse sources, ensuring accuracy in identifying relevant studies and variables. In the synthesis stages, the technology needs to support complex statistical computations while ensuring methodological rigor. In the reporting phase, automation tools must generate clear, interpretable results that comply with reporting standards. At each stage, TTF is applied to examine how effectively technologies meet these task requirements.
- *TTF across AMA stages:* TTF emphasizes the alignment of technology with the specific tasks to be performed. In AMA, this approach ensures that automation technologies remain well-suited to the requirements of each task, leading to improved performance and greater user satisfaction.

The application of TTF in AMA allows for a richer understanding of how automation technologies can improve efficiency, accuracy, and user satisfaction. For instance, while UTAUT may evaluate whether researchers will intend to use automated tools in MA, TTF assesses whether these tools will improve accuracy and reduce time and labor requirements. Specifically, TTF in AMA helps enable evaluation across three critical dimensions: data quality assessment (reliability of automated extraction), system effectiveness (enhancement of the MA process), and user satisfaction (accessibility across expertise levels). This analytical framework has therefore been used in this study to share insights for optimizing AMA tools, advancing task–technology research, and improving user experience and performance outcomes.

### 2.3 Research questions

Having laid out the background of history and evolution of MA, this article applies TTF constructs to better inform on aspects related to AMA deployment, such as the current approaches in use, challenges being faced, future trends, and the overall impact on evidence synthesis. We provide a comprehensive review of the development of AMA over the past decade. Our review highlights how various tools are being applied across different disciplines and how they have developed over time to provide a comprehensive understanding of AMA in evidence synthesis. Accordingly, we have posed four research questions that will be addressed in this review. These are:

- *RQ1 (Descriptive)*: What are the current landscape and key characteristics of AMA approaches?
- *RQ2 (Analytical)*: How does our analytical framework illuminate the strengths and limitations of current AMA approaches within each information processing stage?
- *RQ3 (Comparative)*: What are the distinct patterns in AMA implementation, effectiveness, and challenges observed across medical and non-medical domains?
- *RQ4 (Future-oriented)*: What are the critical gaps and future directions for AMA development, and what obstacles need to be addressed to realize its full potential for evidence synthesis?

## 3 Methodology

This section details the methodologies that provide a prelude to the review process and for the presentation of our results. The review follows the PRISMA criteria in providing answers to the four research questions. Next, we introduce our information processing-centric model to evaluate the alignment between AMA tools and the specific tasks they are designed to support.

### 3.1 PRISMA process

Our investigation of automation in MA followed PRISMA guidelines, employing a systematic search strategy. The database search was restricted to “MA” terms to enhance precision. Broader terms, such as “systematic review,” were not included, as pilot testing indicated they mainly retrieved irrelevant records. Accordingly, the initial search employed the string (“meta-analysis” OR “meta analysis”) AND (automation OR automated OR “machine learning” OR “artificial intelligence” OR AI OR “natural language processing” OR “large language model” OR LLM), with title, abstract, and keyword field filters applied where available (e.g., TITLE-ABS-KEY in Scopus; [Title/Abstract] in PubMed), across PubMed, Scopus, Google Scholar, IEEE Xplore, and ACM Digital Library databases. This presented a preliminary overview of research activities within the stated field of interest, offering a broad yet comprehensive summary of the general characteristics of MA prevalent in existing literature. Next, we established clear inclusion criteria and practical constraints: (1) published from January 2014 to August 2024; (2) focus on explicitly MA-specific automation tools; (3) full-text availability with sufficient methodological detail (e.g., at least four pages with technical descriptions); and (4) shorter records (e.g., abstracts and posters) were excluded due to insufficient information and empirical or quantitative evaluations in automation techniques of MA. Table 1 details the eligibility criteria. Furthermore, to enhance coverage and overcome potential oversights from database-centric searches, we conducted bidirectional citation chaining “snowball” methods.^44^ ^,^ ^45^ This involved both backward tracing (reviewing references cited in the retrieved studies) and forward tracing (identifying later works citing the selected papers) through Scopus and Google Scholar, expanding our temporal scope to 2006–2024. This approach not only expanded coverage by incorporating relevant gray literature and emerging frameworks but also established thematic linkages between foundational methodologies and their contemporary implementations.

**Table 1:** Criteria for study selection using PRISMA

	Inclusion criteria	Exclusion criteria
**Study design**	Studies describing or evaluating computational tools or AI-based or machine learning methods applied to at least one stage of the MA process	Manual-only MAs without any automated tools or software (e.g., traditional narrative reviews conducted through manual screening^46^)
**Technology**	Use of automation tools (e.g., text mining, machine learning models, and natural language processing) in MA processes	Studies primarily focused on theoretical discussions of MA without applying any automation tools (e.g., conceptual frameworks proposed without tool development or testing^47^)
**Data sources**	MA studies incorporating datasets from multiple sources (e.g., PubMed, Google Scholar, Scopus, etc.)	
**Evaluation metrics**	Studies evaluating the performance of AMA tools, ideally using quantitative metrics (e.g., accuracy, efficiency, and time savings). Inclusion is allowed for studies using qualitative metrics if justification for relevance is provided.	Studies applying automation tools without evaluating their performance (e.g., descriptive applications without reporting accuracy, efficiency, or other metrics^48^)
**Publication type**	Peer-reviewed journal articles, conference papers, and preprint articles	Gray literature, opinion pieces, editorials, or conference abstracts without full methodological detail (e.g., institutional reports, theses, opinion or commentary articles, journal editorials, or short conference abstracts without accompanying full papers^49^)
**Language**	Articles published in English	
**Time limit**	All publication dates from 2006 to 2024 were accepted.	Duplicates of the same study.
**Outcomes**	Studies discussing efficiency, reliability, or scalability improvements in MA due to automation	Studies focused on outcomes unrelated to the impact of AMA (e.g., substantive research findings or methodological discussions unrelated to AMA^50^)

**Figure 1:**
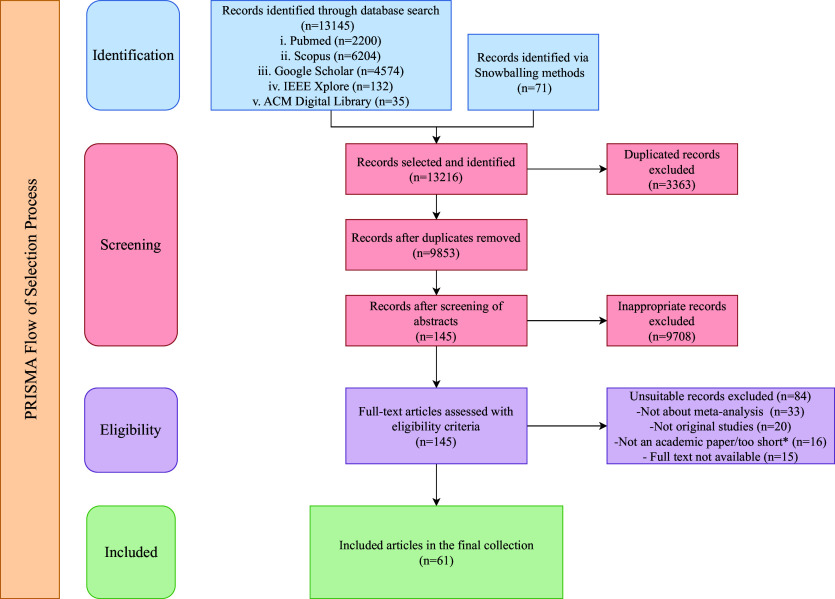
PRISMA workflow. *Note*: “Too short” = records with fewer than four pages, excluded for lacking methodological detail.

**Figure 2:**
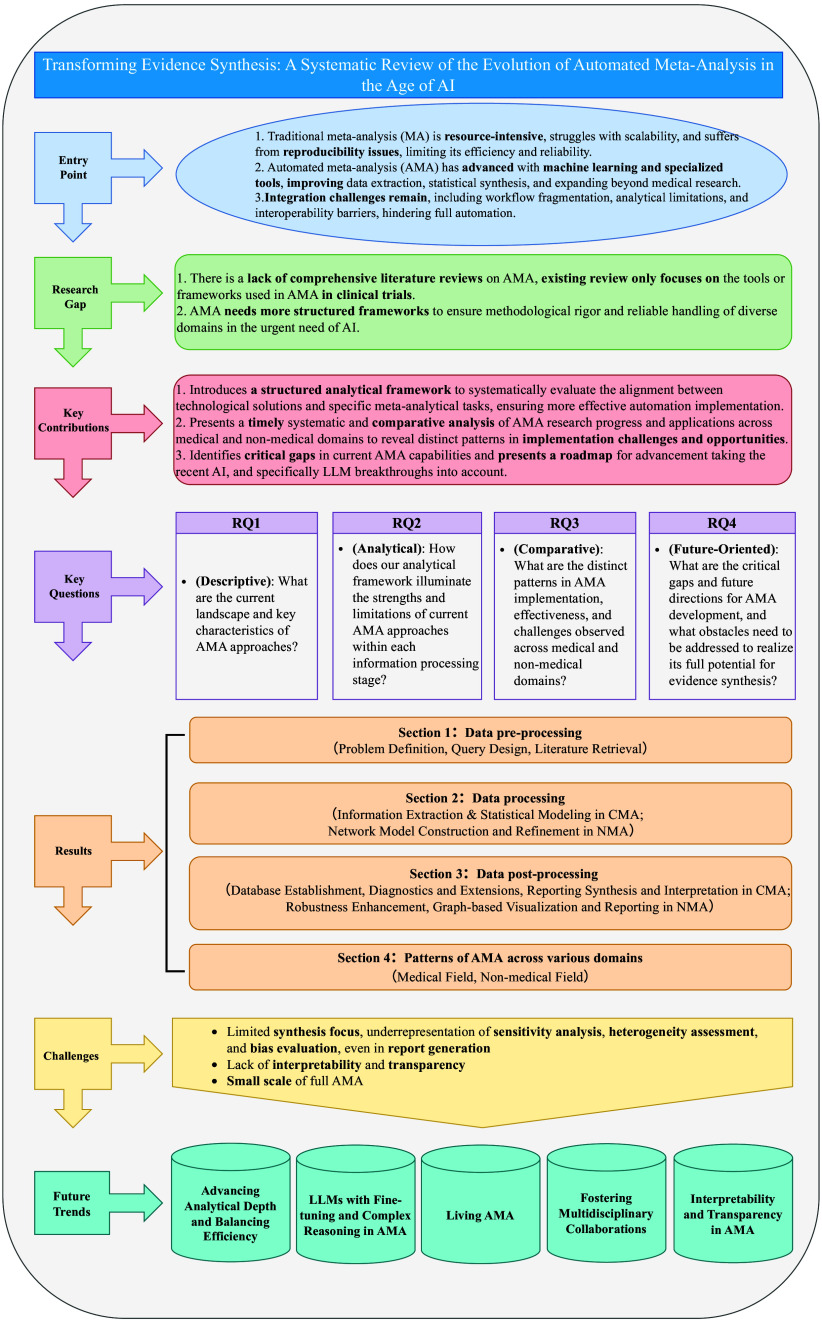
Holistic framework for this review.

The systematic review process, managed through Zotero 7, began with duplicate removal followed by a two-phase screening. One reviewer (L.L.) conducted the initial screening of titles and abstracts to exclude irrelevant records, while two additional reviewers (A.M. and T.S.) independently checked and confirmed the results. The same procedure was applied for the full-text review and any discrepancies in selection were resolved through consensus discussions among the reviewers. The PRISMA flowchart (shown in Figure 1) details the selection process. Data were analyzed and narratively summarized, with descriptive statistics presented in tables or graphs based on each study’s aim. This process identified 13,145 initial studies (2,200 PubMed, 6,204 Scopus, 4,574 Google Scholar, 132 IEEE Xplore, 35 ACM Digital Library, and 71 snowball), which were refined to 61 studies (see the Supplementary Material) after removing 3,363 duplicates and excluding 9,708 studies through screening. The whole visual illustration of our systematic review (shown in Figure 2) outlines the key steps, addresses four research questions, highlighting key contributions, current challenges, and future trends in AMA.

### 3.2 Progressive phase structure in TTF

AMA streamlines the traditional resource-intensive process of MA by integrating automation for data extraction, analysis, and synthesis, enhancing efficiency while reducing human error and statistical expertise requirements. MAs can be categorized along multiple dimensions, including network structure, data type, statistical framework, update approach, and purpose. Among these, this review focuses on widely used and methodologically distinct approaches: conventional MA (CMA) for direct pairwise comparisons and network MA (NMA) for integrating both direct and indirect evidence across multiple interventions.

**Figure 3:**
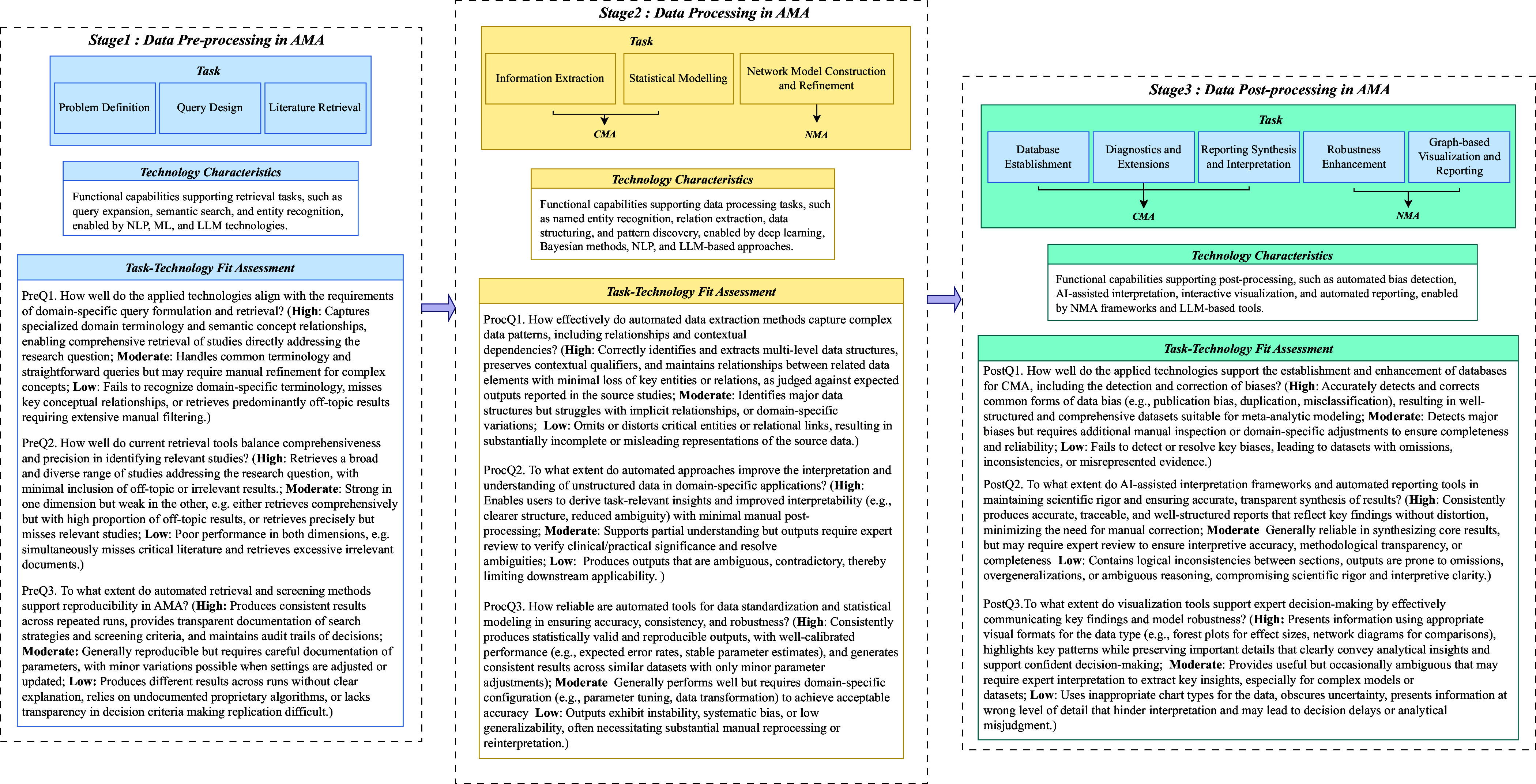
Progressive phase structure with TTF model. *Note*: “PreQ”, “ProcQ”, and “PostQ” denote analytical questions from the pre-processing, processing, and post-processing stages, respectively. Assessment ratings (H = High, M = Moderate, L = Low) are defined above.

While MA methodologies (e.g., the Cochrane Handbook) provide a comprehensive set of methodological stages, these frameworks were not originally designed with automation in mind. Existing automation tools target isolated stages of the process; however, there remains no overarching framework to systematically organize and align automation efforts across the full MA workflow. To address RQ1 (What are the current landscape and key characteristics of AMA approaches?), we introduce the progressive phase structure (PPS), an automation-oriented framework that complements existing methodologies by structurally organizing automation tasks across the entire process. Figure 3 illustrates the PPS framework, which categorizes automation processes into three distinct phases across both CMA and NMA:

- *Pre-processing stage:* Encompasses problem definition, query design, and literature retrieval. NLP, ML, and LLMs can significantly reduce time spent on these labor-intensive tasks.
- *Processing stage:* Involves information extraction (IE) and statistical modeling in CMA or network model construction and refinement in NMA. Automated tools leveraging NLP, ML, and LLMs help extract required datasets and other relevant information and achieve high efficiency.
- *Post-processing stage:* Focuses on database establishment, diagnostics, and reporting in CMA, and robustness enhancement and visualization in NMA. Different automation tools can enhance reproducibility through standardized reports and dynamic visualizations, thereby improving transparency.

PPS does not aim to replace existing methodological taxonomies but to provide a complementary, automation-oriented structure. It organizes the workflow into three high-level phases that correspond to established MA tasks outlined in the Cochrane Handbook (see Table 2), ensuring methodological completeness while offering a streamlined and automation-compatible perspective on the review process. To assess automation effectiveness, we also integrate the TTF model with PPS, providing a structured approach to evaluating alignment between specific MA tasks and available automation tools. This approach systematically deconstructs the automation process into granular components and assesses technological fit at each stage, which operationalizes this alignment by defining:

- *Tasks:* Fundamental, well-defined tasks performed at each PPS phase (e.g., problem definition, query design, and literature retrieval). These tasks represent the concrete units of analysis for evaluating technology alignment.
- *Technology characteristics:* Functional capabilities (e.g., IE, document classification, and text generation) enabled by current automation tools, such as NLP models, ML algorithms, and LLMs.
- *TTF assessment:* Structured evaluation questions designed to systematically assess the degree to which available automation tools support the defined tasks, using a three-level qualitative scale (high/moderate/low).

**Table 2:** Task-level mapping between PPS automation phases and Cochrane methodological stages

PPS phase	Core automation tasks (PPS)	Corresponding Cochrane methodological stages
Pre-processing	Problem definition, query design, and literature retrieval	Question formulation; eligibility criteria definition; search strategy development; literature search; and study screening and selection
Processing	Information extraction, statistical modeling (CMA), or network model construction and refinement (NMA)	Data extraction; assessment of risk of bias; pairwise meta-analysis (CMA); and network meta-analysis (NMA)
Post-processing	Database establishment, diagnostics and reporting (CMA), and robustness enhancement and visualization (NMA)	Interpretation of results; sensitivity analysis; certainty of evidence (e.g., GRADE); and reporting and visual presentation of findings

The three-level TTF assessment questions were developed through iterative pilot coding and team discussions to enhance conceptual clarity and ensure coverage across all AMA stages. For the actual application of these assessments, one reviewer (L.L.) initially extracted data from each included study (e.g., tool name, application stage, functionality, methodological approach, reported performance, and limitations). Two additional reviewers (A.M. and T.S.) independently checked and confirmed the extracted information. The extracted data were then mapped to the PPS framework. TTF ratings were assigned using the same process: L.L. provided initial judgments based on the predefined scale (H-high, M-moderate, and L-low), and A.M. and T.S. independently reviewed the ratings. Any discrepancies in data extraction or assessment were resolved through structured consensus discussions. By structuring AMA through PPS and rigorously applying the TTF model (all details were fully provided in Figure 3), this framework provides a robust methodological foundation for evaluating automation effectiveness in AMA.

## 4 Results

**Figure 4:**
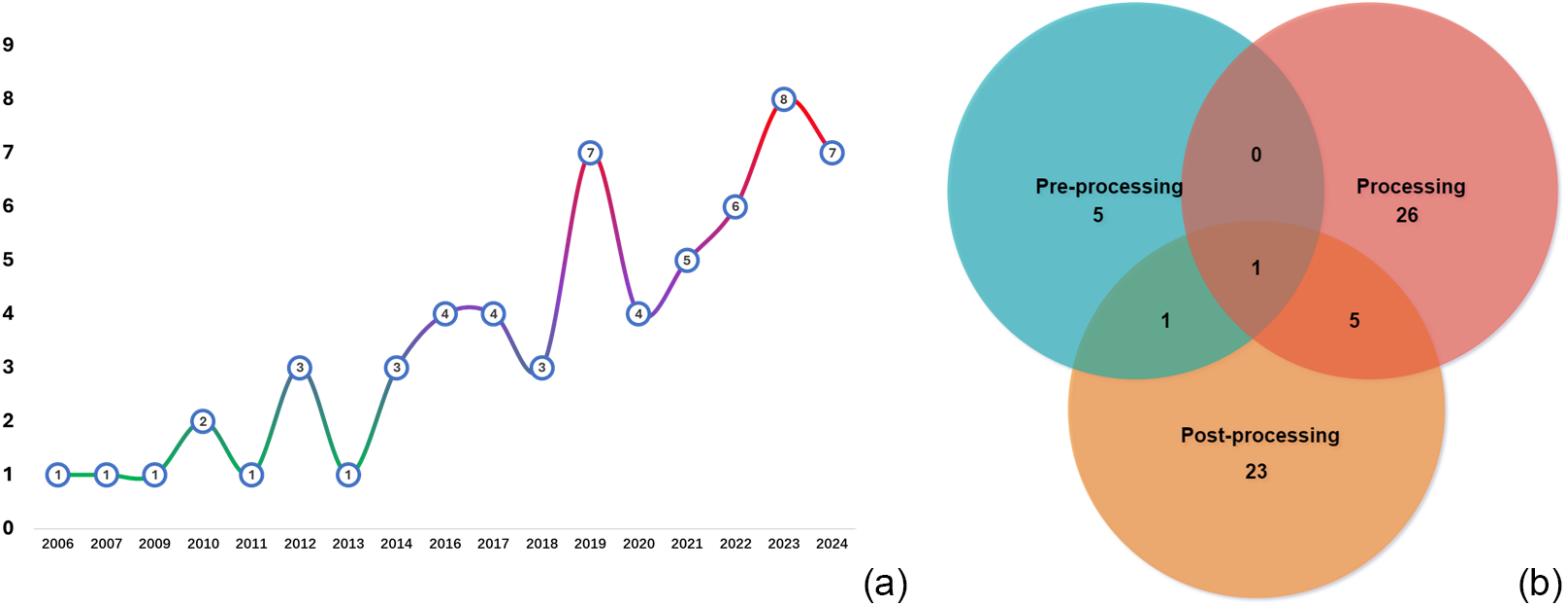
Temporal patterns in AMA publications (a) and proportional discrepancies across different stages (b).

Our review identified AMA publications primarily from journals (72%), conferences (25%), and preprints (3%). Figure 4(a) illustrates the temporal trends showing growth from a single publication in 2006–2009 to seven in 2024. This acceleration, particularly from 2018 onward, coincides with broader AI advancements and increased availability of computational resources. Despite the growth, the relatively low publication volume indicates AMA remains an emerging field with substantial exploration potential. Besides, analysis of PPS implementation revealed that 89% of studies focused on automating a specific MA step, while only 11% addressed multiple stages. Notably, just one study (2%) attempted full integration across all MA stages, highlighting a significant methodological gap. This indicates that while isolated automation tools have advanced considerably, creating seamless multi-stage workflows remains challenging. Figure 4(b) shows that the processing stage dominates AMA research efforts. This concentration likely stems from the technical feasibility and maturity of NLP and ML tools for IE. As IE represents a fundamental prerequisite for all MAs, automation in this area yields substantial efficiency gains. In contrast, the later MA stage involves complex, context-dependent synthesis, which raises further automation challenges, limiting the broader adoption of the system throughout the process.

To address RQ2 (How does our analytical framework illuminate the strengths and limitations of current AMA approaches within each information processing stage?), we provide a comprehensive task breakdown aligned with our analytical framework (refer Figure 3). Automation requirements and success rates vary significantly due to differences in data structure, synthesis models, and computational complexity. Our analysis examines automation strategies and tools employed in both CMA and NMA, identifying distinctive characteristics in each approach. The following sections detail automation processes across PPS stages within the TTF model.

### 4.1 Automation of data pre-processing

Pre-processing in MA comprises problem definition, query design, and literature retrieval, which are all critical for refining datasets for subsequent analysis. The quality of research questions and query design directly influences the relevance and comprehensiveness of retrieved literature. With increasing data volumes, automation has become essential for managing meta-analytic datasets while minimizing bias, as MA performance fundamentally depends on the retrieved literature. In this study, automation is defined for each stage of the meta-analytic workflow. In pre-processing, it refers to algorithmic or rule-based methods that minimize human involvement in problem formulation, query design, and literature retrieval. Our review shows that AMA studies on pre-processing have been developed and tested only within pairwise (CMA) frameworks. Although the procedures are conceptually identical for CMA and NMA, no studies have explicitly adapted automation to accommodate NMA-specific practical considerations, such as capturing all relevant interventions and comparators so that studies form a connected treatment network. Accordingly, current automation efforts in pre-processing remain limited to the CMA context. Table 3 presents a structured evaluation of studies focused on automating this phase, applying TTF to assess alignment between technologies and specific pre-processing tasks.

**Table 3:** Task–technology fit assessment for pre-processing in AMA

Study	Task	Technology characteristics	Task–technology fit assessment
Bosco et al.^51^	Query design and literature retrieval	MetaBUS search engine implementing a taxonomy-based hierarchical keyword structure and Boolean logic to support structured data acquisition	**Fit:** PreQ1=H; PreQ2=M; PreQ3=L **Pro:** Aligns search queries with a predefined taxonomy of 194 constructs, enhancing consistency and reducing human error in keyword formulation **Con:** Requires manual taxonomy updates and lacks adaptability to new or cross-domain terms, with no audit trail to ensure reproducibility.
Xiong et al.^13^	Literature retrieval	Machine learning (K-means clustering with maximum entropy classification) for PubMed text features	**Fit:** PreQ1=H; PreQ2=H; PreQ3=H **Pro:** Machine learning reduced manual screening workload by 87% (from 4,177 to 555), while identifying exactly the same 29 studies as manual selection, demonstrating high precision and recall. **Con:** The supervised model required an initial manually labeled training set, which may limit full automation in different domains and necessitates expert input during setup.
Yang et al.^52^	Literature retrieval	API-based E-utilities programming with XML parsing	**Fit:** PreQ1=M; PreQ2=M; PreQ3=H. **Pro:** Established an API-integrated E-utilities framework that automatically harvested and parsed PubMed XML data with transparent parameters and reproducible workflows, tested on the topic of “the relationship between aspirin drugs and myocardial infarction,” where it retrieved 32 documents. **Con:** Retrieval quality depended on fixed keyword queries, lacking semantic expansion or adaptive tuning to optimize recall–precision tradeoffs.
Deng et al.^53^	Literature retrieval	NLP-assisted MeSH query expansion	**Fit:** PreQ1=H; PreQ2=H; PreQ3=M. **Pro:** Reduced abstract-screening workload by 84% (2,774 vs. 16,941 abstracts) with 93% automated coverage (132/142 studies), which improved to 99% (141/142) after manual reference review across ten validated meta-analyses. **Con:** Reproducibility depends on domain-specific lexical dictionaries, limiting cross-disease generalization and requiring manual updates for new terminologies.
Wei et al.^54^	Query design and literature retrieval	LLM (GPT 3.5)-assisted semantic query normalization	**Fit:** PreQ1=H; PreQ2=H; PreQ3=H. **Pro:** Trained and evaluated on the dataset containing a total of 13,460 studies, improved retrieval accuracy for unstructured queries by 55.93% in Dice and 56.83% in mIoU through deterministic ChatGPT-based semantic augmentation. **Con:** Despite robust within-domain performance, generalization to non-neuroscience terminologies remains limited.
Ghanaati et al.^55^	Query design and literature retrieval	LLM (GPT-3.5 Turbo) screening via API-driven PICOS-guided retrieval	**Fit:** PreQ1=M; PreQ2=H; PreQ3=H. **Pro:** Completed screening of 1,198 abstracts within 1 hour, achieving 95% sensitivity, 99% negative predictive value, and 40%–83% workload savings compared with physicians. **Con:** Query formulation relied on manually designed broad PICOS keywords without adaptive semantic expansion, limiting domain-specific retrieval precision across topics.
Luo et al.^56^	Query design and literature retrieval	LLM (GPT-3.5 Turbo) integrated with LangChain and retrieval augmented generation (RAG)	**Fit:** PreQ1=H; PreQ2=H; PreQ3=M. **Pro:** Achieved 81%–87% accuracy, up to 95% NPV and 0.43–0.39 MCC across 24,534 studies, demonstrating high retrieval precision and efficiency with reproducible LangChain–RAG configuration. **Con:** Despite strong quantitative results, retrieval performance remained sensitive to minor prompt wording changes.

*Note*: PreQ = pre-processing question (1: alignment with domain-specific query requirements; 2: balance of comprehensiveness and precision; and 3: reproducibility of automated retrieval and screening).

Traditional MA requires researchers to craft search strategies that balance breadth and specificity, an inherently complex process that is dependent on researcher expertise.^12^ Automation tools have progressively reshaped this stage. Early systems, such as MetaBUS and MeSH-based expansion frameworks, standardized query formulation within domains, capturing up to 99% of eligible studies while reducing screening workloads by over 80%.^51^ ^,^ ^53^ ML approaches, exemplified by K-means classification, achieved comparable precision to manual screening while drastically cutting dataset size.^13^ LLM-based methods further advanced retrieval efficiency and comprehensiveness, reporting sensitivities up to 95% and workload reductions of 40%–83%.^54^ ^–^ ^56^ Despite these gains, reproducibility remains a limiting factor, whereas ML and LLM pipelines, though reproducible within fixed configurations, are sensitive to domain shifts and prompt variation. Overall, automation in pre-processing in CMA has evolved from static keyword expansion to semantically enriched retrieval, improving coverage and efficiency but still lacking in cross-domain generalizability and standardized reproducibility.

### 4.2 Automation of data processing

Our review highlights the critical role of automation in the data processing phase for both CMA and NMA methodologies. While both approaches aim to enhance meta-analytic efficiency and reliability, they involve distinct automation requirements. CMA prioritizes IE and statistical modeling for synthesizing individual study data, whereas NMA focuses on network model construction and refinement, addressing challenges in inconsistency detection and network connectivity assessment. The following sections provide an in-depth examination of these tasks and their automation potential.

#### 4.2.1 Automated data processing in CMA

Following the pre-processing stage, the next critical task in CMA is IE and statistical modeling. IE techniques transform unstructured text into analyzable, structured data—a fundamental prerequisite for CMA. Key subtasks include named entity recognition (NER) for identifying critical variables and relation extraction (RE) for determining relationships between entities across research articles. Automated IE substantially reduces manual data entry, although performance often varies by domain complexity and terminology. For statistical modeling, automation increasingly relies on algorithmic and workflow-level standardization, enabling reproducible computation of effect sizes and model estimation across meta-analytic datasets. However, model selection must align with specific data types and research objectives: some statistical frameworks offer specialized capabilities for particular data structures (e.g., binary or time-to-event outcomes), while others provide broader applicability across diverse datasets and contexts. Table 4 summarizes studies on automating the data processing phase in CMA using the TTF model, assessing alignment between technologies and tasks requirements while providing insights into their effectiveness and limitations.

**Table 4:** Task–technology fit assessment for automated data processing in CMA

Study	Task	Technology characteristics	Task–technology fit assessment
Michelson^16^	NER+RE for RCTs	Rule-based pattern matching with heuristic classification	**Fit:** ProcQ1=H; ProcQ2=M; ProcQ3=M **Pro:** Achieved 100% precision extracting treatment/control ratios across four verified studies, preserving relational structure for odds-ratio computation. **Con:** Recall limited to 26.7%, with semantic ambiguity in endpoints (e.g., remission vs. relapse) reducing interpretability and reproducibility.
Boyko et al.^57^	RE in vaccine trials (e.g., dendritic cell vaccination)	Semi-supervised graph-based and RandomForest modeling	**Fit:** ProcQ1=H; ProcQ2=H; ProcQ3=M. **Pro:** Extracted 927 patient-group objects from 71 studies (F1 0.6–1.0) enabling structured causal feature mining. **Con:** Manual corpus annotation, ontology extension, and user correction remain required, limiting end-to-end automation and large-scale reproducibility.
Neppalli et al.^58^	NER for quantitative variables in STEM	Supervised Naïve Bayes classifier with bag-of-words context representation and k-fold cross-validation (MetaSeer.STEM)	**Fit:** ProcQ1=H; ProcQ2=M; ProcQ3=M **Pro:** Achieved precision 0.896 and recall 0.786 (F1 0.829) on labeled reliability corpus, showing accurate numeric variable identification across 100 studies. **Con:** Manual annotation and lack of table parsing limit contextual completeness and domain transfer, constraining the generalizability of extracted numeric relations.
Lorenz et al.^59^	RE for IPD MA	Rule-based logic regression with simulated annealing optimization for automatic variable allocation	**Fit:** ProcQ1=H; ProcQ2=M; ProcQ3=M **Pro:** Applied to 34 heterogeneous cohorts, achieving mean sensitivity 0.80 and specificity 0.70 (PPV 0.34, NPV 0.95) in validation; demonstrated reproducible performance for automated variable harmonization across clinical and population studies. **Con:** Manual rule definition for each target variable and low PPV in validation necessitate human oversight and limit end-to-end automation and scalability.
Yang et al.^52^	NER for clinical outcome variables in PDFs	Rule-based PDF parsing and table reconstruction	**Fit:** ProcQ1=H; ProcQ2=M; ProcQ3=M **Pro:** Achieved 100% extraction accuracy for structured tables across tested PDFs (seven related clinical trials); correctly populated variables for downstream meta-analytic computation. **Con:** Extraction limited to tabular data with formatting defects; absence of semantic validation and manual R integration reduce interpretability and reproducibility.
Devyatkin et al.^60^	NER+RE in cell-based immunotherapy	Hybrid rule-based and ML framework combining gazetteers, syntax–semantic parsing, and Eclat co-occurrence mining	**Fit:** ProcQ1=H; ProcQ2=M; ProcQ3=M **Pro:** Applied to 96,160 PubMed abstracts, achieving precision 0.95/recall 0.73 (disease), 0.96/0.52 (cell), 0.50/0.77 (cell-role); syntax–semantic features substantially improved extraction quality over lexical baseline. **Con:** ML post-filtering module was not fully implemented; relation extraction remained corpus- and rule-dependent, limiting reproducibility.
Pradhan et al.^61^	Clinical NER in ClinicalTrials.gov	Rule-based structured pipeline using EXACT engine with XPath parsing and structured XML mapping	**Fit:** ProcQ1=H; ProcQ2=M; ProcQ3=M **Pro:** Extracted 1,571 quantitative data fields from 173 trials with 100% accuracy in field mapping and consistent output for downstream meta-analytic models. **Con:** Context-insensitive field mapping and lack of semantic or unit-level validation require manual review, limiting full automation and cross-domain robustness.
Cheng et al.^20^	NER+RE for causal variable and effect extraction	Deep-learning pipeline combining BERT-based entity tagging, dependency parsing, and causal graph modeling with automated edge validation	**Fit:** ProcQ1=H; ProcQ2=H; ProcQ3=M **Pro:** Achieved F1 = 0.88 for causal variable detection and 0.82 for relation labeling across 12K PubMed abstracts, enabling consistent causal graph construction. **Con:** Limited generalizability beyond medical abstracts due to domain-specific embeddings and the absence of uncertainty calibration reduce reproducibility across heterogeneous corpora.
Anisienia et al.^62^	NER+RE for research method classification in IS	Deep transfer learning (ULMFiT) multilabel text classifier with self-supervised language model and fine-tuning	**Fit:** ProcQ1=H; ProcQ2=M; ProcQ3=M. **Pro:** ULMFiT model achieved P = 0.74, R = 0.64, F1 = 0.66, and Exact Match = 0.91, surpassing prior SVM and GloVe baselines on a corpus of 5,388 IS papers. **Con:** Class imbalance and domain-specific pretraining constrain generalizability; long-document truncation and lack of explainable reasoning limit interpretability and full automation.
Alisa et al.^63^	NER+RE in Cytology	Hybrid rule-based + ML method combining morphological-syntactic parsing (ISA-NLP), FastText embeddings, and ontology-driven	**Fit:** ProcQ1=H; ProcQ2=M; ProcQ3=M **Pro:** Achieved P = 0.92–0.96 and F1 = 0.51–0.66 (in 330 PubMed Central abstracts) for cell-type entities using syntactic–semantic features and ontology-guided role assignment, substantially outperforming the baseline. **Con:** Limited recall (R = 0.38–0.51) and manual corpus expansion dependence restrict coverage and generalizability across new biomedical domains.
Donoghue and Voytek^64^	NER+RE for Event-related potential pattern	Dictionary-based entity recognition and co-occurrence relation extraction	**Fit:** ProcQ1=H; ProcQ2=M; ProcQ3=M **Pro:** Extracted 10,558 ERP articles and identified 98 ERP components, 38 cognitive, and 24 disease terms; fully automated co-occurrence analysis produced structured relational matrices linking components and domains. **Con:** Reliance on manually defined dictionaries and exclusion-word rules introduces noise and limits semantic depth.
Mutinda et al.^65^	NER+RE in breast cancer RCTs	BERT-based PICO entity recognition with UMLS-assisted normalization and rule-based event relation extraction	**Fit:** ProcQ1=H; ProcQ2=H; ProcQ3=M **Pro:** Achieved F1=0.83 on PICO extraction across 1,011 RCT abstracts; automatic mapping to UMLS ensures domain-consistent structured output. **Con:** Model struggles with multi-arm or complex sentence structures, limiting cross-domain reproducibility despite strong task-specific accuracy.
Mutinda et al.^17^	NER+RE in breast cancer RCTs	BioBERT-based PICO sequence labeling with rule-based numeric relation parsing and acronym expansion	**Fit:** ProcQ1=H; ProcQ2=H; ProcQ3=M **Pro:** Achieved F1 up to 0.91 for participant extraction and 0.81 for outcomes on 600 PubMed RCT abstracts, demonstrating high structural accuracy and domain alignment. **Con:** Limited corpus size and reliance on handcrafted numeric rules hinder cross-domain reproducibility and full-text generalization.
Zhang et al.^66^	RE in TCM splenogastric RCTs	VBA-Excel integration with meta-evidence database	**Fit:** ProcQ1=H; ProcQ2=H; ProcQ3=M **Pro:** Established standardized Excel templates (122 fields) with VBA-driven validation and seamless integration to the meta-evidence database. **Con:** Automated extraction is achieved via structured templates and VBA scripts, limiting generalization to unstructured sources.
Kartchner et al.^67^	NER+RE in clinical RCTs	LLMs (GPT-3.5 Turbo and GPT-JT-6B) with zero-shot prompting	**Fit:** ProcQ1=H; ProcQ2=H; ProcQ3=M **Pro:** Demonstrated fully automated zero-shot IE from RCT abstracts (ReMedy and CML database) to extract PICO elements and quantitative outcomes with field-level accuracy (e.g., cancer type: 0.90 accuracy; CML phase: 0.92 Jaccard), and reliably detecting when information was missing. **Con:** Requires post-processing to normalize verbose or inconsistent outputs and remains limited by abstract-only inputs and hallucination risk.
Shah-Mohammadi and Finkelstein^68^	NER+RE in clinical RCTs	Zero-shot GPT-3.5 prompting for XML-to-SQL conversion and structured table reconstruction	**Fit:** ProcQ1=H; ProcQ2=H; ProcQ3=M **Pro:** Achieved 97% accurate extraction of tabular outcome data (means, SDs, and cohort sizes) from PubMed XML, evaluated on three clinical trial papers. **Con:** Prompt sensitivity and untested generalizability across diverse clinical table structures.
Yun et al.^69^	NER+RE in RCTs	Eight different LLMs (GPT-4, GPT-3.5, Alpaca 13B, Mistral 7B Instruct v2, Gemma 7B Instruct, OLMo 7B Instruct, PMC LLaMA, and BioMistral)	**Fit:** ProcQ1=H; ProcQ2=M; ProcQ3=M **Pro:** Compared eight LLMs, with GPT-4 achieving 100% precision extracting treatment/control ratios in four verified RCTs, preserving relational structure for odds-ratio computation. **Con:** GPT-4 recall only 26.7%, while smaller or biomedical models underperformed, with semantic ambiguity and inconsistent outputs reducing reliability.
Wang and Luo^70^	NER+RE in educational systematic reviews	Few-shot prompting with hierarchical schema	**Fit:** ProcQ1=H; ProcQ2=H; ProcQ3=M **Pro:** Achieved near-human precision, recall, and F1 ( 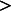 90%) on 20 PICO data elements using hierarchical extraction and semantic reasoning. **Con:** Evaluation limited to a small dataset (32 studies) and single-domain educational context, leaving cross-domain robustness and large-scale generalizability untested.
Choi et al.^71^	Statistical modeling	Bayesian hierarchical mixture model estimated via Markov Chain Monte Carlo with an alternative EM-based maximum likelihood implementation for computational acceleration	**Fit:** ProcQ1=H; ProcQ2=H; ProcQ3=M **Pro:** Introduced a probabilistic latent-variable framework unifying heterogeneous microarray data via POE transformation, achieving cross-study (three studies) gene signature reproducibility and external prognostic validation (C-index up to 0.75). **Con:** Assumes study-level homogeneity; MCMC is computationally intensive and EM approximation shows reduced stability under high inter-study variance, limiting robustness in large-scale omics integration.
Marot et al.^72^	Statistical modeling with small sample sizes	Hierarchical mixture model combining moderated effect sizes with inverse-normal *P*-value aggregation, implemented in the metaMA R package	**Fit:** ProcQ1=H; ProcQ2=H; ProcQ3=H **Pro:** Particularly suitable for small-sample microarray datasets, where variance-shrinkage moderation achieves high sensitivity (up to 26.7%) and stable detection of differentially expressed genes; *P*-value combination further enhanced gene ranking consistency (AUC 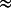 96.6%). **Con:** Effect-size methods are more conservative and less sensitive than *P*-value combinations, trading detection power for stricter FDR control and requiring prior cross-study normalization for comparability across datasets.
Viechtbauer^73^	Statistical modeling	Comprehensive R framework implementing fixed-, random-, and mixed-effects models with multiple heterogeneity estimators (REML, DL, ML, and EB) and moderator analysis via weighted least squares	**Fit:** ProcQ1=H; ProcQ2=H; ProcQ3=H **Pro:** Flexible for integrating data from multiple sources; supports various data types, but computationally demanding for large-scale datasets. **Con:** Lacks real-time analysis of very large datasets; not optimized for speed in high-dimensional data.
Willer et al.^74^	Statistical modeling in large-scale genomic MA	Dual-strategy framework combining sample-size-based	**Fit:** ProcQ1=H; ProcQ2=H; ProcQ3=H **Pro:** Achieves near-linear scalability and full automation for summary-statistic meta-analysis of millions of SNPs with high speed and scalability. **Con:** Limited flexibility for non-genomic data types; primarily optimized for SNP-level GWAS results and not readily adaptable to other biological domains.
Wang et al.^75^	Statistical modeling	Unified R framework (MetaOmics) integrating effect-size, *P*-value, and rank-based models (MetaDE) with hybrid pathway-level aggregation (MetaPath) for differential expression and enrichment analysis	**Fit:** ProcQ1=H; ProcQ2=H; ProcQ3=H **Pro:** Combines twelve meta-analysis algorithms and three pathway models with robust variance weighting, adaptive weighting, and rank-based statistics; achieves high sensitivity and reproducibility in multi-study genomic integration. **Con:** Focused on microarray data; limited support for next-generation sequencing and other omics types.
Suurmond et al.^76^	Statistical modeling for subgroup and moderator analyses	Spreadsheet-based framework	**Fit:** ProcQ1=H; ProcQ2=H; ProcQ3=M **Pro:** Offers an easy-to-use tool for subgroup and moderator analysis in Excel, ideal for researchers and educators without programming experience. **Con:** Limited to the DerSimonian–Laird estimator and single-covariate meta-regression; lacks advanced models (REML, multivariate, or SEM-based meta-analysis).
Debray et al.^77^	Statistical modeling of diagnostic and prognostic models	Unified framework integrating discrimination (c-statistic), calibration (O:E ratio and slope), and heterogeneity estimation across binary and time-to-event outcomes, implemented in the metamisc R package	**Fit:** ProcQ1=H; ProcQ2=H; ProcQ3=H **Pro:** Comprehensive approach for evaluating predictive models with incomplete data; ideal for diagnostic and prognostic studies. **Con:** Complex analysis requiring strong statistical knowledge may not be accessible for non-expert users.
Dockès et al.^78^	Statistical modeling to predict brain activity patterns	Machine-learning framework combining TF–IDF semantic encoding, non-negative matrix factorization, and adaptive ridge regression	**Fit:** ProcQ1=H; ProcQ2=H; ProcQ3=H **Pro:** Valuable for neuroimaging analysis; comprehensive predictive framework linking 13,459 studies and 7,547 neuroscience terms; accurately generalizes to rare or unseen concept combinations (median 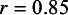 , AUC=0.90). **Con:** Limited to neuroimaging data, not applicable to other fields or generalizable to broader biological analysis.
Peñaloza^79^	Statistical modeling in logical	Mathematical model for combining proportions and estimating trait prevalence	**Fit:** ProcQ1=H; ProcQ2=H; ProcQ3=M **Pro:** Introduces a rigorous logical language to represent and combine confidence intervals, enabling automated reasoning over statistical results and consistency checking across studies. **Con:** Framework remains theoretical and limited to independent binomial intervals; lacks implementation for large-scale or non-binomial data integration.
Llambrich et al.^80^	Statistical modeling for non-integral data	*P*-value and fold change integration	**Fit:** ProcQ1=H; ProcQ2=H; ProcQ3=H **Pro:** Enables quantitative meta-analysis using only *P*-values and fold-changes **Con:** Accuracy limited by missing variance estimates and heterogeneous detection platforms; not applicable to omics data with raw effect-size structures.
Lu et al.^81^	Statistical modeling	Random-effects and multivariate regression framework for cross-study microbiome inference	**Fit:** ProcQ1=H; ProcQ2=H; ProcQ3=H **Pro:** Combines REML-based random-effects meta-analysis with multivariate regression to control heterogeneity and reveal consistent microbial associations across studies. **Con:** Dependent on predefined covariate structures and limited modeling flexibility for longitudinal or compositional data.

*Note*: ProcQ = processing question (1: extraction of complex patterns and contextual relationships; 2: enhancement of structure and interpretability of unstructured data; and 3: reliability of automated standardization and statistical consistency).

**Table 5:** Task–technology fit assessment for automated data processing in NMA

Study	Task	Technology characteristics	Task–technology fit assessment
Van Valkenhoef et al.^82^	Construction and refinement of network models	Algorithmic framework generating Bayesian hierarchical random-effects consistency models via minimum-diameter spanning tree parametrization	**Fit:** ProcQ1=H; ProcQ2=H; ProcQ3=H **Pro:** Enables analysis of large and complex treatment networks while improving efficiency and reproducibility by minimizing subjective decisions in model specification. **Con:** High computational demand, particularly for large and dense treatment networks.
Van Valkenhoef et al.^83^	Construction and refinement of network models (automated detection of inconsistencies)	Node-splitting method for identifying discrepancies between direct and indirect evidence	**Fit:** ProcQ1=H; ProcQ2=H; ProcQ3=M **Pro:** Enables systematic assessment of inconsistency across all treatment comparisons; improves efficiency and reproducibility by automating model generation and reducing ambiguity in multi-arm trial parameterization. **Con:** High computational demand due to estimation of multiple node-splitting models; residual ambiguity between heterogeneity and inconsistency requires manual interpretation.
Thom et al.^84^	Construction and refinement of network models (network connectivity analysis)	Graph-theory-based	**Fit:** ProcQ1=H; ProcQ2=H; ProcQ3=H **Pro:** Reduces manual workload in connectivity assessment; improves confidence in indirect comparisons. **Con:** Limited to binary connectivity and step-count metrics.

*Note*: ProcQ = processing question (1: extraction of complex patterns and contextual relationships; 2: enhancement of structure and interpretability of unstructured data; and 3: reliability of automated standardization and statistical consistency).

*Information extraction:* Across reviewed studies, automation in IE has evolved from rule-based NER toward deep learning and LLM-driven RE. Early NLP pipelines, such as EXACT,^61^ achieved 100% data accuracy and a 60% reduction in extraction time, demonstrating high data precision and complete structural mapping. However, reliance on domain-specific vocabularies constrained generalization, limiting interpretability beyond predefined contexts. Hybrid and deep learning methods, such as BERT-based PICO extraction, improved both structural accuracy and semantic clarity, though they still required carefully curated training data to ensure consistent interpretation across studies.^17^ ^,^ ^65^ More recent LLM-based frameworks, such as MetaMate^70^ and GPT-3.5-powered systems,^67^ ^,^ ^68^ further enhanced contextual linking and conceptual coherence. However, they show only moderate reliability due to sensitivity to prompts and inconsistent XML parsing. Overall, IE automation has advanced in structural precision and interpretability, but continues to face challenges in achieving stable, cross-domain reproducibility.

*Statistical modeling:* Automation in statistical modeling primarily enhances computational reproducibility and standardization. Early probabilistic frameworks^71^ ^,^ ^72^ automated effect-size estimation and variance moderation, achieving high precision and structural completeness and strong interpretability in small-sample analyses. However, assumptions of study homogeneity and computational intensity limited robustness in large-scale applications. R-based automation tools like *metafor*,^73^ METAL,^74^ and *MetaOmics* ^75^ established computational pipelines, incorporating parameter and varied heterogeneity estimators, resulting in high reproducibility. These systems ensured reliable model fitting across datasets but still required domain expertise to ensure valid inferences. Subsequent tools, including *metamisc*,^77^ AMANIDA,^80^ and NeuroQuery,^78^ extended automation to predictive and cross-domain contexts. These frameworks integrated heterogeneous data types and automated statistical evaluations, achieving high data precision and semantic interpretability while maintaining reproducibility through transparent algorithms and standardized output. Overall, statistical modeling automation now provides stable and fully reproducible computational workflows, supported by R-based and algorithmic frameworks. Nonetheless, model selection, sensitivity analysis, and interpretation of complex heterogeneous data remain reliant on expert judgment.

#### 4.2.2 Automated data processing in NMA

In NMA, constructing and refining network models represents a critical challenge distinct from CMA. While CMA primarily extracts information and develops statistical models, NMA must integrate both direct and indirect evidence across multiple interventions through complex network structures. Automation in this domain enhances model consistency, computational efficiency, and reduces manual intervention. Table 5 provides an overview of the included studies in NMA through the TTF model. Van Valkenhoef et al.^82^ pioneered this progress by developing a Bayesian consistency model generation framework that transformed what was previously a manual process requiring subjective parameter decisions. Extending this work, they introduced an automated node-splitting procedure for systematic inconsistency detection, further strengthening analytical transparency and comparability across treatment networks, though reproducibility was moderately limited by residual ambiguity between heterogeneity and inconsistency.^83^ More recently, Thom et al.^84^ applied graph-theoretical metrics to automate network connectivity analysis, reducing manual workload and reinforcing confidence in indirect comparisons across complex networks. Collectively, these advances reducing researcher subjectivity and enabling reproducible, scalable evaluation of increasingly intricate treatment networks.

### 4.3 Automation of data post-processing

Having examined automation in data pre-processing and processing for both CMA and NMA, we now focus on data post-processing, a critical phase involving result refinement and synthesis to ensure reporting accuracy and clarity. This increasingly important area of AMA research enhances analytical precision and advances evidence synthesis. The following sections will provide a detailed characteristics of CMA and NMA post-processing automation.

#### 4.3.1 Automated data post-processing in CMA

Our examination categorizes CMA automated post-processing into three domains: (1) database establishment for structured data organization; (2) diagnostics and extensions for bias or heterogeneity assessment; and (3) reporting synthesis and result interpretation for standardized findings presentation. In this context, automation refers to the use of computational systems (e.g., AI-assisted reporting frameworks and visualization tools) to perform data consolidation, interpretive synthesis, and result presentation with minimal human intervention. Table 6 presents the TTF alignment for CMA post-processing studies.

**Table 6:** Task–technology fit assessment for automated data post-processing in CMA

Study	Task	Technology characteristics	Task–technology fit assessment
Yarkoni et al.^85^	Database Establishment	MySQL-based text text-mining	**Fit:** PostQ1=H; PostQ2=M; PostQ3=L **Pro:** Constructed a database of 3,489 studies and 100,953 foci with 84% sensitivity and 97% specificity. **Con:** Limited by lexical coding approaches and inconsistencies in brain coordinate reporting, reducing mapping accuracy and reliability.
Feichtinger et al.^86^	Database Establishment	R and MySQL	**Fit:** PostQ1=H; PostQ2=M; PostQ3=L **Pro:** Integrated 80 curated datasets from 45 experiments across 13 cancer types with standardized QC criteria and uniform normalization, ensuring reproducible structured data. **Con:** Dependent on Affymetrix platform and public repository availability, excluding newer cancer types and lacking cutting-edge methodologies, limiting applicability to recent research.
Feichtinger et al.^87^	Database Establishment	MySQL and web technologies	**Fit:** PostQ1=H; PostQ2=M; PostQ3=L **Pro:** Established local databases integrating UniGene, Ensembl, and HGNC with quality filters excluding low-count and mixed libraries, enabling structured profiles across 36 tissues. **Con:** Restricted to EST repositories and limited tissue coverage; excludes emerging RNA-seq datasets, constraining updates and applicability to current research.
Michelson^16^	Result synthesis and interpretation	Paule–Mandel random-effects modeling	**Fit:** PostQ1=M; PostQ2=M; PostQ3=L **Fit:** Successfully computed overall treatment effects using random-effects synthesis, producing logically summaries, validated on ten studies comprising 2,926 samples. **Con:** Depends on basic clustering, overlooking advanced methods like meta-regression or subgroup analyses, interpretation limited by small-scale validation.
Shashirekha and Wani^88^	Database Establishment	R and MySQL	**Fit:** PostQ1=H; PostQ2=M; PostQ3=L **Pro:** Integrated multiple GEO microarray studies using GPL files through automated R-based normalization and quality filtering, enables non-experts to conduct complex gene expression analyses across platforms. **Con:** Restricted preprocessing techniques and limited dataset availability may preclude the inclusion of detailed or large-scale genomic studies.
Craig et al.^89^	Diagnostics and extensions	Web crawlers and NLP	**Fit:** PostQ1=H; PostQ2=H; PostQ3=L **Pro:** Integrated inference engines with hypothesis-exploring ontologies to extract and aggregate effect sizes, supporting automated bias detection and cross-study consistency checks. **Con:** System complexity limits accessibility for users without expertise in web crawlers and NLP, lacks uncertainty visualization.
Yang et al.^52^	Result synthesis and interpretation	R-*Meta* package	**Fit:** PostQ1=H; PostQ2=H; PostQ3=M **Pro:** Streamlined the MA process by providing a user-friendly framework for applying various statistical methods. Facilitates integration of multiple datasets efficiently, validated on seven clinical trials. **Con:** Limited scalability and user interactivity; lacks advanced visualization or sensitivity diagnostics.
Hu et al.^90^	Result synthesis and interpretation	Clustering methods without parameter tuning	**Fit:** PostQ1=H; PostQ2=H; PostQ3=M **Pro:** Effectively applied across ten heterogeneous cytometry studies totaling 2,926 samples, enabling the identification of common cell populations. **Con:** Limited in detecting rare cell populations, potentially missing critical insights.
Ryu and Wendt^91^	Result synthesis and interpretation	Integrating METAL and GCTA module	**Fit:** PostQ1=H; PostQ2=H; PostQ3=M **Pro:** Achieved 66% improvement in differential protein detection over single studies and maintained stable integration accuracy (IDR=90.83%) across simulated and empirical proteomics datasets. **Con:** Effect-size interpretation limited to aggregated *p*-values; lacks model-based heterogeneity visualization and variance decomposition for complex experimental designs.
Lam et al.^92^	Result synthesis and interpretation	R-based platform	**Fit:** PostQ1=H; PostQ2=H; PostQ3=H **Pro:** Performs imputation QC, association testing, and meta-analysis with bias diagnostics via LD score regression, generating publication-ready forest and QQ plots for over 800 consortium datasets. **Con:** Dependent on pre-defined imputation panels and limited user control over internal weighting models, reducing flexibility for non-standard data structures.
Cheng et al.^20^	Diagnostics and extensions in detecting bias	Causal inference techniques	**Fit:** PostQ1=H; PostQ2=H; PostQ3=M **Pro:** Introduced causal graphical diagnostics to identify and adjust confounding in effect-size estimation, enhancing bias detection and cross-study robustness verification. **Con:** Users must interpret causal graphs manually, Assumes single-ignorability and is restricted to RCTs, reducing versatility for observational studies.
Ngo et al.^93^	Result synthesis and interpretation	Joint text–image representation learning using transformer encoding and 3D CNN regression	**Fit:** PostQ1=H; PostQ2=H; PostQ3=M **Pro:** Provides a powerful tool for large-scale neuroimaging research, synthesizing brain activation maps with improved accuracy over traditional methods. **Con:** Effect-size interpretation remains opaque due to non-linear embeddings, potentially leading to mapping inaccuracies.
Sabates et al.^94^	Reporting synthesis and result interpretation	Web-based platform	**Fit:** PostQ1=H; PostQ2=H; PostQ3=M **Pro:** Provides a user-friendly semi-automated platform that generates pooled Hedges’ g with confidence and prediction intervals, forest and funnel plots, and GRADE-based evidence summaries. **Con:** Requires accurate input coding and manual study inclusion decisions.
Burgard et al.^95^	Reporting synthesis and result interpretation	R-based metafor/metaviz pipeline, YAML-controlled reporting, and open API integration for continuous meta-analytic updates	**Fit:** PostQ1=H; PostQ2=H; PostQ3=H **Pro:** Generates dynamic meta-analytic reports with standardized forest and funnel plots, continuously updated effect sizes and heterogeneity estimates, ensuring transparent and reproducible evidence dissemination. **Con:** Relies on contributor compliance and metadata completeness, incomplete reporting across studies can reduce synthesis accuracy.
Wei et al.^54^	Reporting synthesis and result interpretation	LLMs integrate with Text2Brain	**Fit:** PostQ1=H; PostQ2=H; PostQ3=H **Pro:** LLMs enhance query flexibility and improve alignment with brain activation distributions, addressing Text2Brain’s semantic redundancy. **Con:** Still constrained by brain coordinate complexity and ambiguous queries, affecting accuracy.
Finnigan et al.^96^	Database Establishment	Python Flask web server	**Fit:** PostQ1=H; PostQ2=H; PostQ3=H **Pro:** Facilitates real-time data updating and provides an accessible biocatalysis analysis platform. **Con:** Limited to biocatalysis, reducing applicability to broader biological and chemical domains.
Bremer et al.^97^	Diagnostics and extensions in detecting bias	Python-based pipeline, PCA-driven outlier detection, and hierarchical clustering	**Fit:** PostQ1=H; PostQ2=H; PostQ3=H **Pro:** Integrates 156,000 GC–TOF MS samples with automated normalization and cross-study effect-size validation, achieving reproducible metabolite trends and bias reduction across 350 datasets. **Con:** Limited interpretability for rare-feature metabolites; quality metrics depend on z-score standardization, which may underrepresent low-abundance variability.
Kale et al.^98^	Diagnostics and extensions	D3-based interactive system for uncertainty diagnostics	**Fit:** PostQ1=H; PostQ2=H; PostQ3=H **Pro:** Enables structured extraction and visualization of epistemic uncertainty using hierarchical models and quantile dotplots, improving bias detection and interpretability across studies. **Con:** Supports only independent-effect models and requires substantial user expertise to operate triage-based workflows, limiting accessibility for non-statistical users.
Lu et al.^81^	Reporting synthesis and result interpretation	R-based automated synthesis	**Fit:** PostQ1=H; PostQ2=H; PostQ3=H **Pro:** Provides unified random-effects modeling, multi-factor regression, and correlation diagnostics with rich visualization tools. **Con:** Limited to amplicon-based studies and dependent on pre-defined databases, restricting flexibility for emerging omics types and customized functional annotations.
Rodriguez-Hernandez et al.^21^	Diagnostics and extensions in reducing heterogeneity	R, Bash, and Python-based platform	**Fit:** PostQ1=H; PostQ2=H; PostQ3=H **Pro:** Standardizes workflow and reduces heterogeneity in over 130 GWAS analyses. **Con:** Lacks details on functionalities and customization options, potentially affecting adoption in specialized GWAS studies.

*Note*: PostQ = post-processing question (1: accuracy and completeness of database construction and bias correction; 2: reliability and transparency of AI-assisted interpretation; and 3: clarity and robustness of visualization for decision-making).

*Database establishment:* Automated database establishment in CMA has evolved from structured text-mining to dynamic web-integrated systems that enhance data accessibility and error correction. Early tools, such as Neurosynth,^85^ achieved strong domain accuracy by constructing a MySQL-based database of 3,489 studies with 84% sensitivity and 97% specificity, but lexical coding inconsistencies limited interpretive precision and reproducibility. CancerMA^86^ and CancerEST^87^ integrating up to 80 curated datasets in oncology from multiple experiments and standardizing normalization pipelines, improving structural reliability but remaining constrained to specific platforms and outdated repositories. Subsequent frameworks, such as ShinyMDE,^88^ enhanced accessibility through R-based integration of GEO studies, while RetroBioCat^96^ further advanced reproducibility via real-time data updating and open-access deployment. Across these systems, automation demonstrated strong error control and data standardization but still required domain-specific validation and frequent manual curation to maintain interpretive consistency and update coverage.

*Diagnostics and extensions:* Automation in diagnostics and extensions has significantly improved bias detection, heterogeneity assessment, and interpretive transparency in CMA. Craig et al.^89^ employed web crawlers and NLP-based inference engines to extract effect sizes and detect cross-study inconsistencies, achieving high domain precision and interpretability, though reproducibility was limited by system complexity and lack of visual diagnostics. Cheng et al.^20^ introduced causal inference diagnostics for bias detection in RCTs, enabling adjustment for hidden confounders but requiring manual causal graph interpretation. Recent frameworks demonstrate marked advances in reproducibility: metaGWASmanager^21^ standardized over 130 genome-wide association study (GWAS) workflows, achieving high stability across analyses, while BiNDiscover^97^ and MetaExplorer^98^ further integrated bias quantification and visualization for metabolomics data, reaching high consistency across 350 datasets. Overall, diagnostic automation now exhibits uniformly high domain precision and interpretive clarity, with reproducibility advancing from low to high through modular, transparent pipeline architectures.

*Result synthesis and interpretation:* Automated result synthesis and interpretation have progressed from conventional random-effects modeling to fully integrated AI- and LLM-driven systems. Michelson et al.^16^ and Yang et al.^52^ laid the groundwork with random-effects and R-*Meta*-based synthesis, achieving moderate to high interpretability but limited reproducibility due to scalability constraints. Subsequent frameworks, such as MetaCyto,^90^ MetaMSD,^91^ and RICOPILI,^92^ improved integration accuracy and automation depth, reaching high reproducibility and interpretive precision across large datasets. In parallel, web-based platforms, such as CogTale^94^ and PsychOpen,^95^ introduced semi-automated reporting pipelines that produced standardized, dynamically updated outputs. Recent advancements, including Text2Brain^93^ Chat2Brain,^54^ have integrated LLMs and visualization modules to enhance semantic synthesis. Overall, CMA post-processing remains dominated by conventional synthesis pipelines. LLM-based synthesis is still in its early exploratory stage, offering conceptual promise but limited practical adoption so far.

#### 4.3.2 Automated data post-processing in NMA

In contrast to CMA, automated post-processing in NMA focuses on two primary areas: (1) robustness enhancement to ensure network model validity and stability and (2) graph-based visualization and reporting to facilitate complex treatment network interpretation. Table 7 presents studies relevant to NMA post-processing.

**Table 7:** Task–technology fit assessment for automated data post-processing in NMA

Study	Task	Technology characteristics	Task–technology fit assessment
Van Valkenhoef et al.^99^	Graph-based visualization and reporting	Bayesian modeling and interactive evidence graph visualization	**Fit:** PostQ1=H; PostQ2=H; PostQ3=H. **Pro:** Makes NMA more accessible to users without deep statistical expertise. **Con:** Limited advanced analytical features for experts; mainly designed for simpler analyses.
Neupane et al.^100^	Robustness enhancement	Comparison of R packages: *gemtc*, *pcnetmeta*, and *netmeta* focuses on Bayesian vs. frequentist methods.	**Fit:** PostQ1=H; PostQ2=H; PostQ3=M. **Pro:** Helps researchers select tools based on their specific needs (e.g., statistical method preference and flexibility). **Con:** Limited guidance for researchers without statistical expertise, as some tools are highly technical.
Owen et al.^101^	Graph-based visualization and reporting	R Shiny–netmeta integration to support visualization	**Fit:** PostQ1=H; PostQ2=H; PostQ3=H. **Pro:** Enables real-time interactive network meta-analysis using *netmeta* with automatic inconsistency diagnostics and intuitive visualization, improving accessibility and interpretability for non-technical users. **Con:** Limited to frequentist models and lacks advanced Bayesian or covariate-adjusted analyses, constraining flexibility for complex or hierarchical treatment networks.
Nikolakopoulou et al.^102^	Robustness enhancement focusing on study bias, uncertainty, heterogeneity, etc.	Simulation-based confidence modeling with contribution matrices	**Fit:** PostQ1=H; PostQ2=H; PostQ3=H. **Pro:** Provides an in-depth, structured approach to assessing NMA credibility, ensuring transparent, quantitative interpretation of complex treatment networks. **Con:** Can be resource-intensive, requiring substantial data input and expert judgment for full evaluation.
Chiocchia et al.^103^	Robustness enhancement focuses on addressing missing data bias	A web-application (ROB-MEN) developed to operationalize the CINeMA dimension	**Fit:** PostQ1=H; PostQ2=H; PostQ3=H. **Pro:** Enhances NMA robustness by addressing missing data, a common issue in systematic reviews. **Con:** Focuses narrowly on missing data, excluding other biases, and may require expert interpretation.
Liu et al.^104^	Graph-based visualization and reporting	*BUGSnet* R package	**Fit:** PostQ1=H; PostQ2=H; PostQ3=H. **Pro:** Makes Bayesian NMA more accessible in social science, allowing for complex data handling and incorporating prior knowledge. **Con:** Requires R programming knowledge, limiting access for those without coding expertise.
Reason et al.^105^	Robustness enhancement	LLMs (GPT-4) and Bayesian modeling	**Fit:** PostQ1=H; PostQ2=H; PostQ3=H. **Pro:** Demonstrates end-to-end automation of NMA, achieving 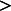 99% accuracy across 20 runs for each case study and strong potential to reduce human labor and error. **Con:** Dependent on LLM consistency and external model calls.

PostQ = post-processing question (1: accuracy and completeness of database construction and bias correction; 2: reliability and transparency of AI-assisted interpretation; 3: clarity and robustness of visualization for decision-making).

*Robustness enhancement:* Automation for robustness enhancement in NMA has evolved from traditional statistical comparisons toward integrated credibility frameworks. Neupane et al.^100^ compared three major R packages—*gemtc*, *pcnetmeta*, and *netmeta*—evaluating their efficiency and flexibility across Bayesian and frequentist models. This comparison achieved high domain fit by clarifying methodological options but required statistical expertise for practical use. Nikolakopoulou et al.^102^ developed CINeMA, a simulation-based framework providing quantitative credibility assessments for complex treatment networks, addressing uncertainty, bias, and heterogeneity. ROB-MEN^103^ automated the evaluation of missing data bias, further strengthening analysis reliability. Most recently, Reaon et al.^105^ explored the integration of LLMs (GPT-4) with Bayesian modeling to achieve near end-to-end automation. However, as in CMA, LLM use in NMA remains preliminary, showing clear potential but lacking solid support at this point.

*Graph-based visualization and reporting:* Graph-based visualization is essential for interpreting complex treatment networks in NMA. Van Valkenhoef et al.^99^ pioneered this direction with ADDIS, a Bayesian graph-based platform that visualized evidence networks and treatment effects through standardized analysis and transparent output. Subsequently, MetaInsight^101^ combined R *netmeta* with a Shiny interface, enabling real-time network visualization, automatic inconsistency diagnostics, and simplified workflows for non-technical users, though limited to frequentist models. Extending accessibility to new fields, Liu et al.^104^ developed BUGSnet, a Bayesian R package designed for psychology and social sciences. Nevertheless, current visualization and reporting systems remain dominated by rule-based and R-based architectures, AI- or LLM-assisted visualization has not yet been applied in NMA practice representing a promising direction for future development.

### 4.4 Patterns of AMA across domains

The PPS with TTF model provides a comprehensive framework for AMA by automating each process step. To answer RQ3 (What are the distinct patterns in AMA implementation, effectiveness, and challenges observed across medical and non-medical domains?), our analysis revealed significant domain-specific variations.

**Figure 5:**
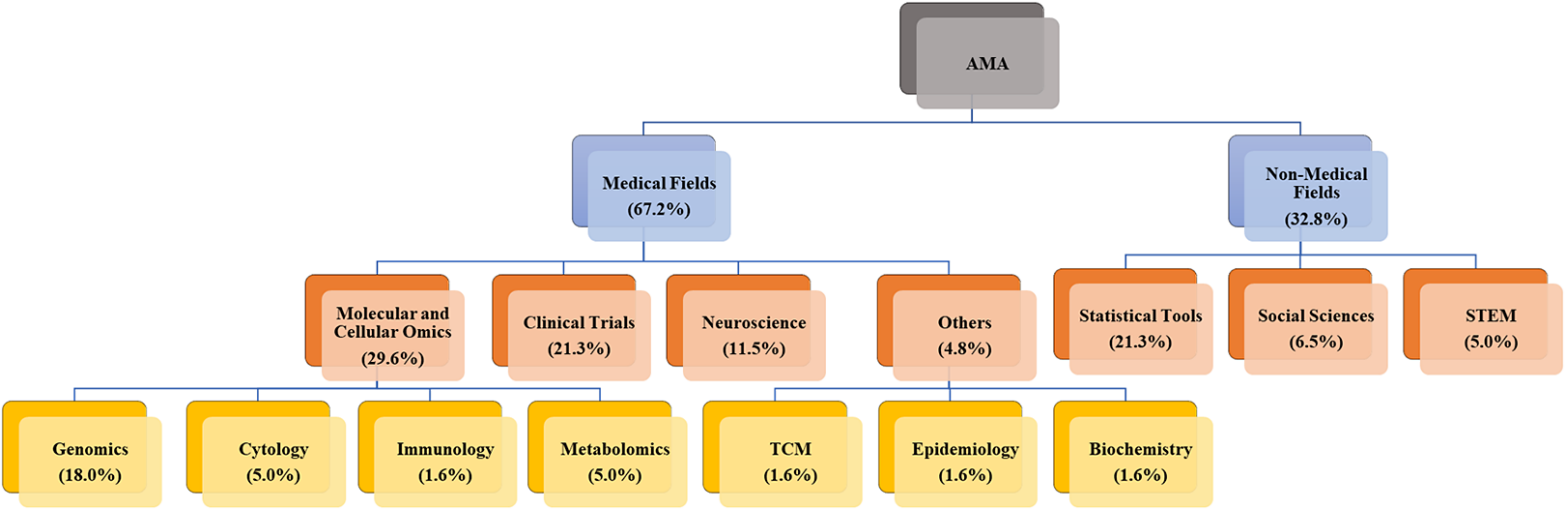
Interdisciplinary applications of AMA across various domains.

A primary distinction lies in the predominant data characteristics and their implications for automation adoption. Medical domains more frequently utilize standardized, structured data from clinical trials, healthcare records, and standardized literature (e.g., CONSORT-compliant reports and structured abstracts). This prevalence of standardization creates a stronger TTF for automated tools, such as NLP, ML, and LLMs, which can efficiently process consistent terminology with minimal human intervention. While medical research certainly includes unstructured elements (such as clinical notes and narrative case reports), the presence of substantial standardized components has enabled earlier and more widespread adoption of AMA. Conversely, non-medical fields (social sciences, management, education, and STEM) predominantly present heterogeneous, less structured data with varied reporting styles and terminologies. Although pockets of standardization exist (e.g., large-scale survey data in the social sciences or structured experimental datasets in STEM), the relative lack of universal standardization frameworks creates greater challenges for current automated tools. This helps explain the lower adoption rates observed in our review (Figure 5). Methodological traditions further differentiate these domains. In the medical domain, established protocols, statistical methods, and data collection guidelines support automation through more predictable, uniform data formats. The greater methodological diversity in non-medical fields, while valuable for exploratory research, complicates the application of automated tools.

**Figure 6:**
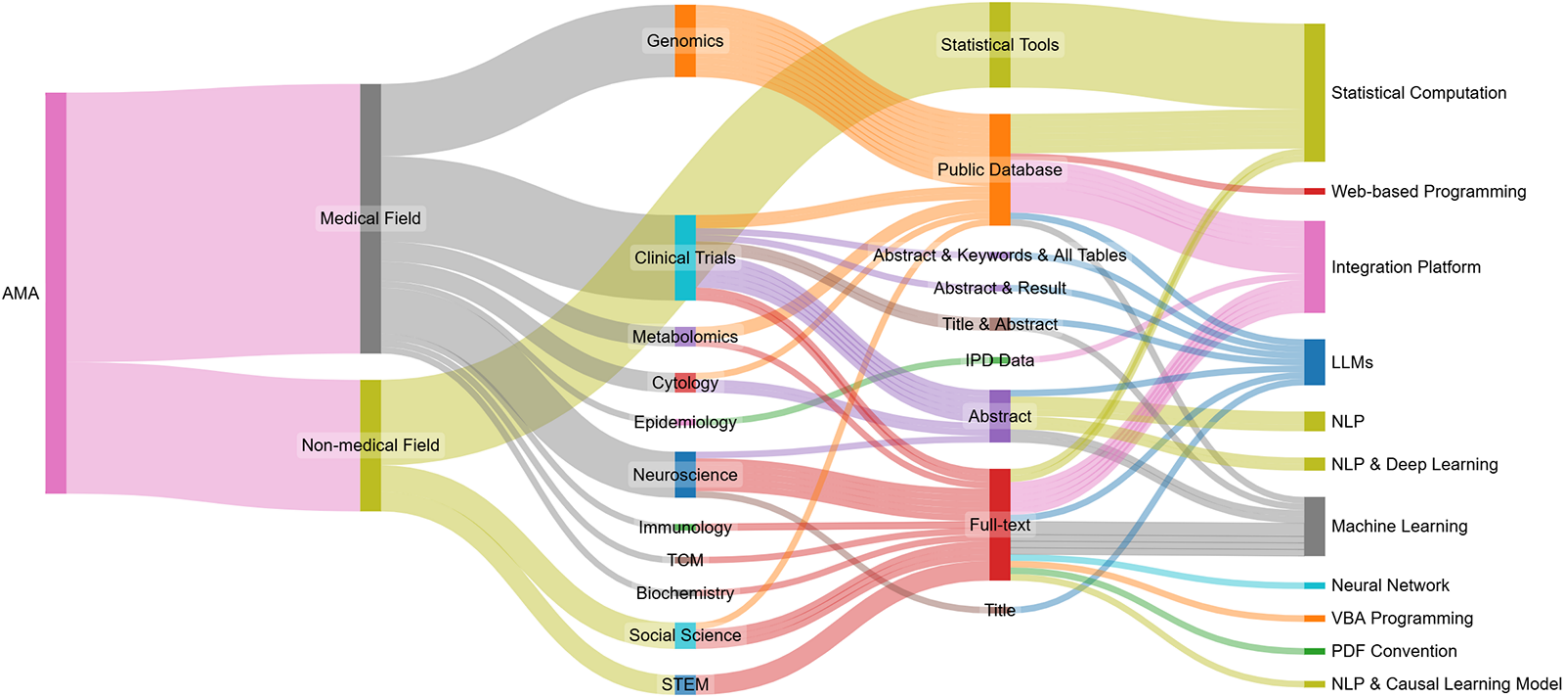
Cross-domain mapping of AMA applications, data types, and methodological approaches.

Our systematic review provides empirical evidence for these domain-specific distinctions. Figure 5 illustrates the disparity: medical applications account for 67.2% of reviewed studies ( 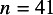 ), compared to 32.8% in non-medical domains ( 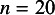 ), with clinical trials alone comprising 21.3% of all AMA implementations. This marked imbalance highlights differences in the maturity of TTF across domains. To complement this proportional view, Figure 6 offers a quantitative mapping of cross-domain linkages among domain applications, data types, and computational methods, with line thickness reflecting application frequency. The visualization reveals a clear pattern: medical AMA primarily draws on structured or semi-structured data sources (e.g., public databases and abstracts) that align closely with NLP- and ML-based automation pipelines. In contrast, non-medical AMA remains centered on statistical and methodological tool development, with social science and STEM studies still at early stages of practical adoption. Taken together, these findings reveal a maturity gradient across domains: medical fields exhibit dense, stable connections between standardized data and well-established computational methods, whereas non-medical domains display more fragmented, exploratory linkages that are still evolving toward systematic automation. Notably, the emergence of LLMs marks a new trend across domains, but their practical applications remain limited in both medical and non-medical fields. Current studies are largely exploratory, focusing on assessing feasibility rather than achieving full automation. Detailed analysis in the following sections illustrates how these differences translate into distinct task–technology alignment dynamics, tool specialization trajectories, and varying levels of automation maturity across medical and non-medical subfields. Importantly, our dual-perspective analysis highlights reciprocal learning opportunities that bridge traditional disciplinary divides. These opportunities for cross-disciplinary collaboration emphasize that advancing AMA requires not only innovation within each domain but also purposeful knowledge exchange across the medical-non-medical divide.

#### 4.4.1 Medical field

AMA in the medical field is rapidly evolving across clinical trials, molecular and cellular omics, neuroscience, and specialized domains.

*Clinical trials:* AMA in clinical trials has progressed from abstract-based data extraction to complex full-text analysis, propelled by NLP, ML, LLMs, and other enhanced computational tools. This development has enabled automation across critical tasks, including literature selection, data extraction, publication bias evaluation, and results synthesis. Early efforts established foundational synthesis capabilities,^16^ while subsequent research improved literature selection^13^ and significantly enhanced data extraction efficiency.^17^ ^,^ ^52^ ^,^ ^61^ ^,^ ^65^ ^,^ ^67^ ^–^ ^69^ Progress also extends to mitigating publication bias.^20^ However, achieving fully AMA remains elusive due to persistent challenges, including data inconsistency, incomplete datasets, and limitations in processing complex full-text content. LLMs also require refinement to interpret intricate analytical demands effectively. Domain-specific applications illustrate both potential and constraints: ChatGPT has been adapted for screening radiology abstracts,^55^ and general LLMs have improved NMA for binary and time-to-event outcomes.^105^ This trajectory highlights AMA’s promise while underscoring the need to address technical barriers for broader applicability. This trajectory illustrates that in structured and protocol-driven clinical environments, automation advances in depth and complexity, yet remains bounded by the limits of text understanding and data completeness.

*Molecular and cellular omics:* This subdomain exemplifies the structured-data and integration-oriented pattern of AMA, where automation can also build on large-scale, standardized repositories and statistical synthesis frameworks. Unlike literature-based clinical trials, omics AMA leverages structured datasets from public repositories like Genevestigator,^106^ GEO, and ArrayExpress,^107^ emphasizing statistical analysis and integration. Key tasks include data processing, multi-omics integration, and differential expression analysis, a range of specialized tools supports these efforts: RankProd,^108^ alongside frameworks by Boyko et al.^57^ and Devyatkin et al.,^60^ facilitates gene expression dataset processing; METAL^74^ enables GWAS MA; ShinyMDE^88^ aids in detecting differentially expressed genes; MetaGWASManager^21^ handles large-scale GWAS data; MetaCyto^90^ analyzes high-dimensional cytometry data; and Amanida^80^ detects study discrepancies. These tools have enhanced data processing and integration, with MetaCyto notably improving efficiency in high-dimensional analysis,^90^ yet challenges like dataset heterogeneity, platform variability, and incomplete data persist. Domain-specific adaptations address some of these issues, such as Amanida’s focus on metabolomics data gaps^80^ and MetaGWASManager’s automation of GWAS analysis,^21^ but broader application remains limited by these constraints. Overall, the trajectory in molecular and cellular omics shows that AMA is still constrained by inter-platform heterogeneity.

*Neuroscience:* The coexistence of quantitative brain-imaging outputs and narrative research reports illustrates the challenge of aligning heterogeneous data modalities within a single automation framework. Neuroscience AMA synthesizes brain-related data using NLP, ML, LLMs, and predictive modeling to identify patterns in cognitive and neural states. This approach targets tasks, such as brain activation mapping, cognitive intervention analysis, and event-related potential (ERP) analysis. Progress in brain mapping tools has evolved from NeuroSynth^85^ to NeuroQuery,^78^ Text2Brain,^93^ and Chat2Brain,^54^ while predictive modeling has advanced through NPDS 0.9^89^ and cognitive intervention repository via CogTale.^94^ Despite these developments, challenges persist, including limited data availability, variability in experimental design, and difficulties in processing large volumes of unstructured text. Domain-specific adaptations, such as probabilistic ERPs literature analysis^64^ and neural networks linking text queries to brain activation in Chat2Brain,^54^ demonstrate potential but highlight the need to address these constraints for broader application. Therefore, this mixed-structure in AMA demonstrates that the coexistence of structured and unstructured data both drives methodological innovation and constrains full automation.

*Specialized domains:* AMA applications extend to specialized domains, including traditional Chinese medicine (TCM), epidemiology, and biochemistry, demonstrating adaptability across diverse research contexts. In these fields, AMA focuses on data processing and evidence synthesis, employing tools, such as logistic regression, ML, and NLP. Notable successes include automated logistic regression for epidemiological individual participant data (IPD) MAs, which reduces processing time and errors.^59^ However, the specialized nature of these systems restricts their generalizability. Domain-specific adaptations, such as TCM literature synthesis for splenogastric diseases^66^ and the RetroBioCat Database for biocatalysis data exploration,^96^ reveal a pattern of constrained generalization. Automation performs exceptionally well in targeted, well-defined contexts but faces challenges when extending beyond these specialized frameworks.

#### 4.4.2 Non-medical field

AMA applications remain limited outside medicine, with only nascent adoption in three key domains: statistical tools, social sciences, and STEM. This scarcity reflects both challenges and opportunities for expanding AMA beyond medical contexts.

*Statistical tools:* This category illustrates a cross-domain methodological pattern of AMA, where automation focuses on enhancing statistical modeling, consistency checking, and computational reproducibility. Although inherently applicable across both medical and non-medical contexts, these tools are typically introduced in the literature as methodological contributions rather than domain-specific applications. For this reason, we present them here at the beginning of the non-medical section. These tools encompass Bayesian random-effects models, graph theory, web-based platforms, and decision rules. Statistical packages, such as *metafor*,^73^ Meta-Essentials,^76^ and *metamisc*,^77^ have improved analysis accessibility, while NMA tools, including *gemtc*, *pcnetmeta*, and *netmeta*,^100^ support complex modeling. Semi-automated systems ADDIS^99^ and analytical frameworks for consistency checks and bias assessment such as Bayesian random-effects models,^82^ ^–^ ^84^ CINeMA,^102^ and ROB-MEN^103^ further refine precision. Web platforms MetaInsight^101^ enhance usability for researchers without extensive statistical expertise. Nonetheless, challenges remain, including limited multi-modal data processing and the growing complexity of modern meta-analytical frameworks. Domain-specific adaptations, such as ADDIS, CINeMA, ROB-MEN, and MetaInsight, address specialized NMA needs but reflect the persistent tension between tool sophistication and broad applicability. Therefore, these developments show a methodological pattern emphasizing statistical rigor and reproducibility.

*Social science:* Social sciences have begun adopting AMA tools for synthesizing diverse data types across disciplines, such as human resource management, psychology, and education, focusing on tasks like data synthesis and predictive modeling. Tools, such as Bayesian methods and LLMs, underpin these efforts. Notable advances include MetaBUS, which streamlines MA across extensive literature volumes^51^; Bayesian NMA opens new possibilities for quantitative analysis^104^; and MetaMate leverages few-shot prompting for data extraction in education.^70^ However, the diversity of data types, particularly qualitative data and complex models, poses significant challenges to automation, highlight the ongoing difficulty of achieving broad applicability across heterogeneous datasets. AMA in the social sciences remains in an exploratory stage, these early attempts mark an important foundation for future integration of LLM-based synthesis, suggesting a gradual but steady shift toward more systematic automation in social research.

*STEM:* AMA in STEM shows progress in literature retrieval and data extraction, leveraging ML-based tools and deep transfer learning. Tools like MetaSeer.STEM^58^ streamlines data extraction from research articles, enhancing literature analysis efficiency, while deep transfer learning systems improve retrieval processes.^62^ AMA adoption in STEM remains in its early stages, with automation primarily targeting specific tasks like information retrieval rather than comprehensive evidence synthesis. The lack of consistent data standards and the wide-ranging diversity of STEM research hinder scalability. However, the presence of structured experimental data makes STEM a promising area for future advancements as methodological and integration frameworks evolve.

## 5 Challenges and future potential for AMA

Despite increasing adoption of AMA techniques, significant challenges remain that must be addressed to realize its full potential for evidence synthesis. To answer RQ4 (What are the critical gaps and future directions for AMA development, and what obstacles need to be addressed to realize its full potential for evidence synthesis?), this section examines key barriers and future directions to enhance AMA’s credibility and utility. These challenges span multiple dimensions: enhancing analytical capabilities while mitigating automation biases; maintaining methodological rigor and transparency; adapting to evolving research technology developments; gaining broader acceptance among stakeholders; and ensuring reliability of synthesized evidence. Prior to presenting our proposed roadmap for AMA development, we assigned values to the “Difficulty” and “Priority” based on a structured methodological framework, grounded in a qualitative assessment of technical, methodological, organizational, ethical, and data-related factors, as well as their anticipated impact on AMA’s development. “Difficulty” reflects the complexity of implementation, considering factors, such as technical barriers (e.g., algorithm complexity and data availability), methodological challenges (e.g., validation rigor), organizational constraints (e.g., interdisciplinary collaboration), and ethical considerations (e.g., transparency). Ratings range from “Low” to “High,” with “Medium” indicating moderate challenges requiring moderate effort or expertise. “Priority” evaluates the urgency and impact of each research direction, integrating immediate practical needs (e.g., addressing current gaps), high-impact potential (e.g., improving validity or scalability), and long-term benefits (e.g., credibility and broad applicability). Ratings are categorized as “Immediate,” “Medium,” or “Long-term,” often combined with qualitative descriptors (e.g., “High Impact and” “Trust”) to reflect multifaceted outcomes. This approach ensures a balanced, evidence-based assessment, and Table 8 presents a prioritized roadmap for AMA development according to this assessment framework. This analysis aims to reveal that advancing AMA requires not only technical innovation, but also methodological refinement and strategic implementation approaches to improve its credibility and utility in diverse research contexts.

**Table 8:** Future research directions for AMA

Category	Future trends	Difficulty	Priority
Advancing analytical depth and balancing efficiency	Sophisticated analytical components: Development and validation of automated algorithms for sensitivity analysis, heterogeneity assessment (including subgroup analysis), bias evaluation (e.g., publication bias and risk of bias), and NMA-specific analyses (e.g., inconsistency detection).	Medium *Methodological & Technical*	Immediate *High Impact & Validity*
	Balancing automation and analytical rigor: Establishing frameworks and best practices to ensure efficient automation does not compromise the depth and methodological rigor of evidence synthesis, requiring human oversight at critical analytical junctures.	Medium *Methodological & Organizational*	Medium *Maintain Credibility & Trust*
	Adapting to diverse input types: Creating flexible AMA systems capable of handling diverse data formats (numerical, text, images, and raw data), necessitating modular architectures and standardized input interfaces.	Low *Technical*	Immediate *Broadened Applicability*
LLMs with fine-tuning and complex reasoning in AMA	Enhanced document analysis: Developing LLMs specifically fine-tuned for analyzing long and complex academic documents, including effective extraction of data from tables, figures, appendices, and supplementary materials, addressing current limitations in context window size and multi-modal data processing.	Medium *Technical & Data Availability*	Immediate *Improved Data Completeness*
	Meta-analytic methodological reasoning and adaptation: Training specialized LLMs to understand and reason about methodological decisions (e.g., fixed vs. random effects models, handling of outliers, adjustment for publication bias, and between-study heterogeneity) in context-sensitive ways.	Medium *Technical & Domain Knowledge*	Immediate *Validity & Trust*
	Multi-step research synthesis: Developing LLMs capable of planning and structuring the entire synthesis process, from questions developments to interpretation of findings, with capabilities to adapt plans based on emerging patterns during data extraction and analysis.	High *Methodological & Cognitive*	Long-term *Process Optimization*
	Transparent LLM decision-making: Implementing XAI techniques to enhance the transparency and interpretability of LLM-driven decisions within AMA workflows, fostering expert validation and building trust in automated outputs, particularly in critical domains like healthcare.	High *Technical & Ethical*	Medium *Increased Trust & Adoption*
	Robust benchmarks and validation: Designing standardized benchmarks and rigorous validation protocols to systematically evaluate the accuracy, reliability, reproducibility, and potential biases of LLM-generated results in AMA, ensuring quality control and facilitating comparative evaluations across different LLM-based tools.	Medium *Methodological & Community Effort*	Medium *Quality Assurance & Comparability*
Living AMA	Dynamic and continuous updating: Developing fully automated “Living AMA” systems capable of dynamic, ongoing updates as new evidence emerges, requiring robust monitoring pipelines, algorithms for reconciling conflicting data, and effective version control mechanisms, moving beyond static, periodic updates.	Difficult *Technical, Methodological & Organizational*	Long-term *Maintain Relevance & Actionability*
Multidisciplinary Collaborations	Fostering trust and effective communication: Building robust multidisciplinary teams encompassing statisticians, computer scientists, domain experts, information specialists, and policymakers, establishing shared goals, standardized workflows, and effective communication channels to overcome disciplinary silos and maximize AMA impact.	Difficult *Organizational & Social*	Long-term *Maximize Impact & Uptake*
Interpretability and Transparency in AMA	Establishing XAI standards and best practices: Developing and disseminating standards and best practices for the integration of explainable AI (XAI) within AMA workflows, focusing on communicating decision-making processes, uncertainty levels, potential limitations, and ensuring responsible and ethical automation.	Difficult *Ethical, Methodological & Community Effort*	Long-term *Ethical & Responsible Innovation*

### 5.1 Advancing analytical depth and balancing efficiency in AMA

A critical and persistent limitation in AMA remains the automation of advanced analytical methodologies, including sensitivity analyses, heterogeneity assessments, publication bias evaluations, and stratified subgroup analyses. While preliminary data processing has advanced significantly, sophisticated analytical automation remains underexplored, compromising the reproducibility and scientific validity of AMA findings. Future research should prioritize three critical areas: (1) Algorithm advancement. Developing frameworks that execute complex analytical functions with minimal human intervention while maintaining methodological rigor, including automated sensitivity analysis and bias detection tools. (2) Methodological balance. Creating frameworks that enhance efficiency without compromising analytical depth and integrity, with strategic human oversight at critical analytical stages. (3) Multi-modal data integration. Incorporating heterogeneous data types (numerical data, medical images, tables, and raw data) through adaptable extraction techniques for comprehensive, statistically sound evidence synthesis. These advancements would elevate AMA beyond basic automation to deliver both sophisticated analytical capabilities and enhanced efficiency, strengthening its credibility in high-impact research domains.

### 5.2 LLMs with fine-tuning and complex reasoning in AMA

LLMs, including those with advanced “thinking” capabilities capable of complex reasoning, offer transformative potential for AMA by efficiently processing unstructured text and extracting critical variables (effect sizes and confidence intervals) from research articles. These “thinking models” in LLMs extend beyond basic data extraction, enabling genuine knowledge synthesis and methodological reasoning, potentially revolutionizing evidence synthesis by integrating statistical and semantic understanding. They function as methodological thought partners, assessing heterogeneity between studies, and adapting analytical strategies to the specific characteristics of included studies, thereby enhancing AMA’s precision, scalability, and adaptability across diverse research contents. However, several challenges hinder their full-scale deployment of LLMs in AMA. These include hallucinations that fabricate results, which is unacceptable in high-stakes applications like healthcare; propagation of implicit biases from training corpora into synthesized outputs; and limitations with extensive context windows when processing journal articles, dissertations, and complex figures/tables.^109^ To maximize this potential opportunity, future research should prioritize developing specialized “thinking LLMs” for analyzing long-form academic content with multi-modal capabilities; enhancing transparency through explainable AI (XAI) techniques to facilitate expert validation of automated extractions; and designing benchmarks and protocols to ensure the accuracy, reliability, and reproducibility of LLM-generated results. These advancements will significantly enhance the reliability and interpretability of LLM-assisted AMA workflows, potentially reshaping the foundations of evidence synthesis methodology and positioning “thinking LLMs” as a cornerstone of future AMA innovation.

### 5.3 Living AMA

Current AMA implementations primarily automate discrete stages of MA but lack mechanisms for continuous, real-time evidence updates. This limitation is particularly evident in Cochrane MAs, which require periodic updates to maintain clinical relevance. A “living AMA” addresses this gap by envisioning a system that can automatically and continuously scan databases for new studies, extract relevant data, and integrate fresh evidence into existing analyses. Realizing this vision should focus on three key aspects. First, designing robust AI-driven mechanisms to identify and validate new studies as they emerge. Second, developing algorithms to make a version control and reconcile conflicting data across studies while preserving analytical transparency. Third, creating efficient alert mechanisms that update researchers without overwhelming them with excessive information. Living AMA approaches have already emerged in related domains, such as “living literature review,”^110^ COVID-19 living MAs,^111^ MetaCOVID project,^112^ and SOLES system.^113^ Building on these foundations, future work must refine the methodological framework for Living AMA to ensure delivery of up-to-date, high-quality evidence synthesis.

### 5.4 Fostering multidisciplinary collaborations

The success of AMA depends on requiring seamless collaborations between statisticians, computer scientists, domain experts, and policymakers. However, interdisciplinary cooperation remains a bottleneck due to differences in methodologies, terminology, and research priorities. Addressing this challenge requires three strategic approaches: (1) interdisciplinary training programs to familiarize researchers with AMA methodologies and computational techniques; (2) joint funding initiatives to support large-scale, collaborative AMA projects; and (3) shared platforms and community to promote cross-disciplinary integration. These approaches leverage complementary expertise: statisticians ensure methodological rigor, computer scientists develop the technical framework, and domain experts provide contextual knowledge to interpret findings meaningfully. Through effective communication and trust-building, AMA can evolve into a widely adopted tool bridging computational power with domain-specific expertise.

### 5.5 Interpretability and transparency in AMA

As AMA tools become more sophisticated, transparency in their decision-making processes becomes increasingly paramount, particularly in high-stakes domains such as medical research where evidence synthesis directly influences clinical decisions. The integration of XAI methods into AMA represents a critical frontier in ensuring credibility and adoption. The challenge of interpretability in AMA extends beyond mere technical performance. While automated systems can significantly reduce the time and effort required for MA, their value diminishes if end-users cannot understand or trust their outputs. This is particularly crucial during the evidence synthesis phase, where complex algorithms process and integrate diverse evidence sources. Recent research^56^ highlights the delicate balance required between efficient automation and maintaining the depth and accuracy of evidence synthesis. Future research should prioritize the standardization of XAI integration within AMA workflows, ensuring automated processes remain transparent, reproducible, and trustworthy. Various XAI techniques, such as rule-based explanations, visual explanations, and sensitivity analysis, may integrate into AMA findings with more accessible and easier adjustments. Through these approaches, AMA can evolve into robust and widely accepted tools that enhance the quality of evidence synthesis.

## 6 Discussion

AMA has emerged as a transformative innovation in quantitative evidence synthesis, driven by exponential growth in literature that demands efficient, scalable, and reproducible quantitative research methods. Advanced AI, particularly “thinking models” with the capable of complex reasoning, has become a cornerstone of this evolution. This review has provided an evaluation of AMA via a descriptive lens (RQ1), analytical lens (RQ2), comparative lens (RQ3), and a future-oriented lens (RQ4). Despite AMA offering significant benefits compared to traditional MA, full automation remains aspirational rather than becoming a standard. This gap underscores the urgent imperative to harness “thinking models,” bridging technical and methodological barriers to position AMA as a critical frontier for future evidence synthesis innovation.

### 6.1 Methodological disparities between CMA and NMA

Quantitative analysis reveals a clear research imbalance, with 81% of AMA studies focusing on CMA versus only 19% addressing NMA. This disparity arises from the inherent complexity of NMA, which requires integrating both direct and indirect comparisons across multiple interventions while accounting for data heterogeneity. CMA, involving primarily pairwise comparisons, is more amenable to automation through established statistical frameworks and increasingly supported by emerging technologies, such as NLP and LLMs. Table 9 highlights key distinctions between CMA and NMA automation. While both CMA and NMA can employ fixed-effects, random-effects, or Bayesian models, CMA analyses are typically simpler in structure and supported by packages, such as *metaMA* ^72^ and *metafor*,^73^ enabling more streamlined automation. In contrast, NMA often involves additional modeling layers to account for indirect comparisons and network consistency, requiring more specialized tools. Visualization in CMA often centers around forest and funnel plots. NMA, while also employing these, additionally requires complex network graphs, inconsistency plots, rankograms, and SUCRA plots, many of which demand manual adjustments or customization. Addressing the NMA automation gap necessitates algorithms capable of handling multi-dimensional, network-structured data while ensuring model transparency and consistency.

**Table 9:** Comparative analysis of CMA and NMA automation

Feature	Conventional meta-analysis (CMA)	Network meta-analysis (NMA)
Data structure	Pairwise comparisons; relatively simple structure, with heterogeneity modeled via random-effects	Multiple interventions; network structure requiring modeling of transitivity and consistency
Statistical models	Primarily fixed-effects and random-effects models; Bayesian approaches also applicable	Fixed-effects, random-effects, and Bayesian models; additional layers for network consistency and indirect comparisons
Automation tools	Statistical packages: metaMA, metafor; supported by emerging technologies, such as NLP and LLMs	Statistical packages: gemtc, netmeta; limited integration with NLP/LLMs to date
Visualization	Forest plots and funnel plots	Forest plots, funnel plots, network graphs, inconsistency plots, rankograms, and SUCRA plots
Automation success	**High** in data extraction due to standardized workflows and simpler data structure, but **low** in synthesis	**Moderate**; limited by network construction, inconsistency modeling, and visualization complexity

### 6.2 Complexity of full automation

Despite notable advancements, full AMA workflow remains elusive. Our review reveals that the majority of existing studies employed semi-automated approaches, with automation largely confined to data extraction and preliminary synthesis. Only one study^52^ has explored full automation across all AMA stages. This highlights a critical gap between the automation of individual components, highlighting the gap between automating individual components and developing integrated, end-to-end systems. Key barriers include: (1) Technical challenges. Data heterogeneity across formats (structured databases, unstructured literature, and high-dimensional biomedical data); computational complexity of advanced meta-analytic methods; and LLM limitations in interpreting context-sensitive statistical details. (2) Methodological barriers. Difficulty automating qualitative judgments (risk-of-bias assessment, confounding adjustment, and evidence grading). (3) Organizational and infrastructural hinders. Limited cross-disciplinary adaptability and absence of universal standards for seamless data integration. Addressing these challenges calls for advancements in AI, carefully designed methodological frameworks, and a clearer, more detailed grasp of how automation demands differ across the AMA workflow. These demands are not uniform but shift significantly across stages. For instance, in Stage 1 (literature retrieval) or Stage 2 (IE), recall is typically prioritized over precision, as omitting relevant studies or variables undermines the comprehensiveness and validity of MA. In contrast, later stages, such as statistical calculation, synthesis, and interpretation, place greater emphasis on accuracy, transparency, and methodological consistency, where overly inclusive results may introduce ambiguity, bias, and analytical distortions. These tradeoffs highlight that what constitutes “optimal” automation is inherently stage-specific and context-dependent. Therefore, we believe that the development of stage-aware benchmarks is an important direction for future research. Importantly, the goal of AMA is not to achieve perfect end-to-end automation but to pursue context-sensitive optimization that enhances analytical utility while preserving scientific integrity.

### 6.3 Ethical and practical considerations

AMA adoption also raises critical ethical questions. One of the most pressing ethical concerns is the risk of bias amplification. AMA systems typically rely on existing datasets, including published literature, trained on existing literature may reinforce systematic publication biases, particularly if they rely on biased data sources. Additionally, the increasing reliance on automation in evidence synthesis introduces concerns about deskilling of researchers, which means as automation takes over certain tasks, researchers may become less proficient in critical appraisal and statistical analysis, potentially reducing the quality of evidence synthesis. Furthermore, the development and adoption of AMA tools are disproportionate, creating risks of global inequity in access to advanced evidence synthesis technologies. If AMA tools remain proprietary, cost-prohibitive, or require specialized technical expertise, low-resource settings may struggle to leverage these innovations, potentially widening disparities in research capacity. Another ethical use of AMA is transparency, without transparency, stakeholder trust in automated evidence synthesis may be undermined, raising concerns about reproducibility and accountability in decision-making. Therefore, researchers looking to adopt AMA should consider that: (1) not all AMA tools are equally effective across disciplines. Choosing the right tool requires an understanding of its strengths, limitations, and adaptability. (2) Rather than seeking full automation, researchers should integrate AMA as an assistive tool while maintaining expert oversight in critical analytical processes. Through these approaches, researchers can balance ethical responsibility, methodological rigor, and transparency without overshadow AMA potential benefits.

### 6.4 Implications for evidence synthesis

The ability of AMA to streamline quantitative evidence synthesis has been widely recognized across biomedicine, neuroscience, epidemiology, and omics research through automating data extraction, statistical modeling, and synthesis processes. Its evolution could significantly enhance efficiency, reproducibility, and scalability. For example, the continued advancement of AMA has the potential to reshape the landscape of evidence synthesis, which enabling more dynamic and responsive updates to existing evidence bases. This could be particularly valuable in rapidly evolving research domains, such as pandemic response or emerging medical technologies. Besides, automation approaches can facilitate data extraction and statistical analysis, thereby minimizing inconsistencies introduced by subjective human interpretation. Large-scale and complex analysis AMA from heterogeneous datasets will extend beyond traditional systematic reviews based solely on published clinical trials.

However, over-reliance on automation without addressing limitations risks undermining synthesized evidence reliability. One of the concerns is the diminished role of expert judgment in study selection, data interpretation, and result contextualization. Besides, many AMA tools operate as black-box systems, making it challenging to trace how decisions—such as study inclusion/exclusion criteria—are made. Moreover, if automation is trained on biased or incomplete datasets, it may affect the accuracy of evidence synthesis, thereby affecting clinical and policy-related decision-making. Furthermore, most existing AMA systems primarily rely on published studies indexed in databases, such as PubMed or Scopus, this focus may reinforce existing publication biases by systematically underrepresenting negative or inconclusive findings, particularly those available only in gray literature, preprints, or non-English sources. Addressing this issue will require developing more inclusive and adaptive search strategies within AMA frameworks.

The emergence of “thinking models” with complex reasoning in advanced AI and LLMs presents a transformative opportunity to revolutionize AMA by bridging computational power with sensitive analytical capabilities. They enable adaptive analytical strategies that can dynamically handle multi-modal datasets, reducing human intervention while maintaining methodological precision. To maximize the benefits of AMA, a balanced and methodologically rigorous approach is therefore essential, integrating “thinking LLMs” while mitigating its inherent challenges. Domain experts should remain actively involved in tasks, such as study selection, risk of bias assessment, sensitivity analyses, and interpretation, ensuring AMA outputs align with established research methodologies. Standardized reporting frameworks should be established to enhance the transparency of AMA methodologies, allowing researchers to audit and validate automated results. More sophisticated statistical modeling techniques and advanced AI techniques should be developed based on data complexity. Finally, as AMA tools become more widely adopted, policymakers should establish clear guidelines in evidence synthesis such as ethical considerations in AI-driven MAs and equitable access to AMA technologies. By integrating these principles and embracing AI-driven breakthroughs, AMA can evolve into a more robust and ethically responsible tool for evidence synthesis, bridging the gap between automation-driven efficiency and the need for methodological rigor and interpretability, and fundamentally transform evidence synthesis across disciplines.

### 6.5 Study limitations

This review is constrained by several interconnected challenges. First, this study’s predominant focus on well-documented tools from literature databases, potentially overlooks other innovative methodologies. Moreover, given the rapid evolution of automation technologies, particularly in artificial intelligence and LLMs, the review’s findings may quickly become outdated without regular updates. The dynamic nature of this field necessitates continuous revision to maintain relevance and usefulness. Second, while the proposed PPS with the TTF model offers a framework to understand AMA development, the criteria for assessing the level of automation remain subjective and qualitative, making it difficult to quantitatively compare the automation capabilities of different tools. Developing more standardized criteria for evaluating these tools would enhance the objectivity and reliability of future reviews. Third, this review is primarily based on the summary and classification of existing literature, without conducting empirical validation or performance evaluation (e.g., comparative experiments of different tools). As a result, some conclusions rely on self-reported findings in the reviewed studies, lacking independent external verification. These limitations highlight the need for ongoing research and development to refine AMA tools, address integration challenges, and ensure that they remain reliable and applicable in diverse research contexts.

## 7 Conclusion

MAs are critical to advancing science. The prospect of automating MAs opens opportunities for transforming quantitative research synthesis and redefining scientific progress. The automation of MAs is desperately needed right now to manage the expanding volume of academic research. We currently stand at the threshold of a significant AI revolution which holds potential to provide solutions to many remaining limitations and unsolved questions in the field of MA automation. To proceed effectively and maximize this opportunity, this study fills the gap in the literature by comprehensively investigating the current landscape of these automation efforts for MAs using a robust framework. This research has assessed existing methodological approaches, compared implementation patterns across various domains, and synthesized key challenges as well as future directions. Our emphasis has been on the potential that increasingly sophisticated LLMs with enhanced reasoning capabilities offer to accelerate progress further. Our research has found that automated tools have excelled at streamlining data extraction and statistical modeling, yet they still remain limited in achieving full-process automation, particularly in advanced synthesis and bias evaluation. Our work finds that future research efforts must prioritize the development of integrated frameworks that not only enhance individual meta-analytic stages but also bridge gaps between them. Efforts need to focus on also refining AI-driven models to improve interpretability and robustness, ensuring that heterogeneous data sources and complex synthesis tasks are effectively managed. Furthermore, standardizing methodologies across disciplines will be essential to unlock the full transformative potential of AMA.

Therefore, as the volume and complexity of academic research continue to escalate, the evolution of AMA represents a pivotal innovation for evidence synthesis. By harnessing advanced AI capabilities and addressing current methodological shortcomings, the research community can significantly enhance the efficiency, accuracy, and reproducibility of meta-analytic practices—ultimately revolutionizing the way we synthesize scientific knowledge.

## Supporting information

10.1017/rsm.2025.10065.sm001 Li et al. supplementary material Li et al. supplementary material

## Data Availability

There is no dataset.

## Footnotes

1
https://training.cochrane.org/online-learning/core-software#RevMan

2
https://www.meta-analysis.com/

3
https://www.stata.com/

4
https://www.ibm.com/spss

## Supplementary material

The supplementary material for this article can be found at https://doi.org/10.1017/rsm.2025.10065.
