# Supplementary material for: Transforming evidence synthesis: A systematic review of the evolution of automated meta-analysis in the age of AI
Source: Res Synth Methods. 2026 Jan 9;17(3):403–50. doi: 10.1017/rsm.2025.10065 (PMC13126215; doi:10.1017/rsm.2025.10065)
Supplement: Li et al. supplementary material [file S1759287925100653sup001.pdf]

Supplementary Material

1 Key Insights from Studies on Automated Meta-Analysis

Table 1: Key Insights from Studies on Automated Meta-Analysis

No	Ref	Core Contribution	Year	Field	Methodology & Purposes	Automated Processing Steps		
						**Pre-processing**	**Processing**	**Post-processing**
1	[19]	RankProd	2006	Molecular and Cellular Omics	Mathematical calculations		※	※
2	[9]	metaArray	2007	Molecular and Cellular Omics	Two general probabilistic models for quantities that are combinable across studies:(1) Markov Chain Monte Carlo techniques (2) expectation-maximization algorithm		※	
3	[30]	metaMA	2009	Molecular and Cellular Omics	Mathematical in effect size calculation and P-value combination		※	
4	[52]	metafor	2010	General Tools	Mathematical calculations		※	
5	[56]	METAL	2010	Molecular and Cellular Omics	Computationally efficient tool for MA of genome-wide association scans: (1) converts the direction of effect and P-value observed in each study into a signed Z-score (2) weights the effect size estimates, or β -coefficients, by their estimated standard errors		※	
6	[59]	NeuroSynth	2011	Neuroscience	Identified psychological terms, extracted brain activation coordinates, mapped brain-cognition links, and decoded constructs using naïve Bayes classification.			※

Table continues on the next page...

No	Ref	Core Contribution	Year	Field	Methodology & Purposes	Processing Steps		
						Pre-processing	Processing	Post-processing
7	[16]	CancerMA	2012	Molecular and Cellular Omics	Raw data from ArrayExpress and GEO repositories underwent manual assessment, quality control with the 'simpleaffy' R package, and pre-processing using standard methods. The data was annotated with databases like Ensembl and HGNC, integrated into the CancerMA website, analyzed using the 'GOstats' R package, and visualized.			※
8	[53]	MetaOmics	2012	Molecular and Cellular Omics	Mathematical calculations		※	
9	[50]	Bayesian model	2012	General Tools	Bayesian homogeneous variance random effects consistency models		※	
10	[51]	ADDIS	2013	Clinical Trials	The unifying data model together with a semi-automated analysis generation system			※
11	[17]	CancerEST	2014	Molecular and Cellular Omics	Obtained the complete data available from the Unigene database, set up a local MySQL database and examined expression profile of submitted genes			※
12	[31]	First attempt of AMA in RCTs	2014	Clinical Trials	Transformed clinical trial abstracts into structured data, groups studies with similar treatments and outcomes, and uses the Paule-Mandel random-effects model to determine the overall treatment effect		※	※
13	[35]	gemtc, pcnetmeta, netmeta	2014	General Tools	Mathematical calculations		※	※
14	[4]	AMA specially in Dendritic Cell Therapy	2016	Molecular and Cellular Omics	Patient-level data on dendritic cell vaccination from 71 studies was semi-supervisedly extracted and transformed into a vector space for analysis and classification.		※	
15	[34]	MetaSeer.STEM	2016	STEM	Automatically converted the PDF into text and manually mark the data of interest, then dataset is used to train classifiers in supervised learning		※	
16	[46]	ShinyMDE	2016	Molecular and Cellular Omics	Multiple statistical methods, including Fisher's, Stouffer's, Minimum p-value, and Maximum p-value, along with their one-sided correction variants		※	

Table continues on the next page...

**No**	**Ref**	**Core Contribution**	**Year**	**Field**	**Methodology & Purposes**	**Processing Steps**		
						**Pre-processing**	**Processing**	**Post-processing**
17	[49]	Node-splitting model	2016	General Tools	Consistency models used in priors and starting values for node-splitting models		※	
18	[3]	MetaBUS	2017	Social Science	A matrix is extracted via OCR, cleaned with VBA scripts, reviewed for errors, and transposed into a standardized format.	※		
19	[10]	Nexus-PORTAL-DOORS System v0.9	2017	Neuroscience	System with components for metadata curation, data retrieval, natural language processing, query expansion, inference extraction, and statistical analysis			※
20	[27]	PROG-IMT	2017	Epidemiology	Manual rules were created for each target variable, optimized with ROC analysis, and used in Boolean logic regression. The simulated annealing algorithm identified the best rule combination to minimize errors.		※	
21	[47]	Meta-Essentials	2017	General Tools	A set of 7 workbooks each designed to serve a special purpose. Each workbook consists of 6 sheets. Mathematical calculations			※
22	[20]	MetaCyto	2018	Molecular and Cellular Omics	Unsupervised analysis and guided analysis to identify common cell subsets			※
23	[57]	robust automatic study selection	2018	Clinical Trials	Publications were grouped using K-means, and relevant clusters identified with maximum entropy classification, followed by manual screening	※		
24	[58]	Automating processes in MA through computer technology	2018	Clinical Trials	Screened in the URL format of PubMed + PMID +XML, extracted table information through smallPDF and analyzed results in R	※	※	※
25	[13]	text analysis in cell-based immunotherapy	2019	Molecular and Cellular Omics	The framework crawled PubMed abstracts, extracted features, identified entities, analyzed relationships, filtered uninformative entities, and mined co-occurrence statistics		※	
26	[11]	metamisc	2019	Clinical Trials	Mathematical calculations		※	
27	[38]	MetaInsight	2019	General Tools	Used R's netmeta and Shiny packages for analysis and user interface			※
28	[40]	EXACT	2019	Clinical Trials	Python library for parsing ClinicalTrials.gov records and an interface for users to specify desired data		※	

Table continues on the next page...

No	Ref	Core Contribution	Year	Field	Methodology & Purposes	Processing Steps		
						**Pre-processing**	**Processing**	**Post-processing**
29	[48]	Stata package"network"	2019	General Tools	Used graph theory to assess the connectivity of evidence networks in network meta-analysis by constructing an adjacency matrix, where rows and columns represent treatments, and entries indicate whether treatments were compared, with zeros on the diagonal.		※	
30	[12]	Semiautomated NLP-based workflow	2019	Clinical Trials	Applied rule-based and NLP-assisted literature screening to identify relevant studies of cancer susceptibility gene penetrance and validated workflow efficiency against traditional manual meta-analysis	※		
31	[43]	MetaMSD	2019	Molecular and Cellular Omics	Integrated multiple mass spectrometry proteomic datasets, standardized quantitative features across studies, and identified reproducible molecular signatures			※
32	[14]	NeuroQuery	2020	Neuroscience	Used a multivariate model trained on 13,459 publications, and inferred semantic similarities across terms using NLP		※	
33	[37]	CINeMA	2020	General Tools	Mathematical calculations			※
34	[24]	RICOPILI	2020	Molecular and Cellular Omics	Integrated PLINK-based QC, EIGENSOFT PCA, EAGLE/SHAPEIT phasing, and IMPUTE2 imputation into a fully automated high-throughput pipeline to harmonize multi-cohort GWAS datasets and accelerate genome-wide association analyses			※
35	[39]	first approach for automated reasoning in meta-analyses	2020	General Tools	Mathematical calculations			※
36	[1]	Information extraction of immunosuppressive cell	2021	Molecular and Cellular Omics	Hybrid approach using dictionaries, a rule-based parser, and a pre-trained machine learning model to identify and filter entities, leveraging external linguistic resources and syntactic-semantic features for entity relationships		※	
37	[2]	Help authors to automate parts of a literature review and mitigate some of the problems associated with everincreasing number of papers	2021	STEM	Used deep transfer learning methods for multi-label classification in recognizing research methods	※		

Table continues on the next page...

No	Ref	Core Contribution	Year	Field	Methodology & Purposes	Processing Steps		
						Pre-processing	Processing	Post-processing
38	[7]	AMA in causal learning perspective	2021	Clinical Trials	Two-step framework: (1) automatic data extraction from publications using NLP, and (2) automatic inference of treatment effects, controlling for biases.		※	※
39	[36]	Text2Brain	2021	Neuroscience	Consists of a transformer-based text encoder and a 3D CNN			※
40	[44]	CogTale	2021	Neuroscience	Three-tier web application with a React-based user interface, a NodeJS backend with a REST API, and an R-based analysis sub-module for statistical analysis and report generation. It uses MongoDB for data storage and integrates with a WordPress site for public access			※
41	[15]	automated meta-analytic tool for ERP-related literature	2022	Neuroscience	Used search terms to gather articles and create data-driven profiles of ERP components, all literature data was collected using the E-utilities API		※	
42	[26]	Amanida	2022	Molecular and Cellular Omics	Mathematical calculations		※	
43	[32]	PICO recognition	2022	Clinical Trials	Developed a BERT-based NER model to extract PICO information from abstracts of breast cancer		※	
44	[33]	AUTOMETA	2022	Clinical Trials	Developed a BERT-based to extract PICO information from abstracts		※	
45	[61]	developed and tested a new form of clinical evidence in TCM	2022	TCM	Created a data sheet for extraction based on Microsoft Excel powered with Visual Basic for Applications, developed TCM database with Python 3.8 and MySQL 8		※	
46	[6]	PsychOpen CAMA	2022	Social Sciences	Built a FAIR-based open platform with R Markdown and Shiny		※	
47	[5]	BinDiscover	2023	Molecular and Cellular Omics	Implemented a PostgreSQL- and Flask-based platform integrating BinBase-processed GC–TOF MS data with machine learning–driven batch correction and statistical meta-analysis			※
48	[22]	MetaExplorer	2023	STEM	Implemented D3.js-based visual analytics with Bayesian modeling to explore and communicate epistemic uncertainty			※

Table continues on the next page...

No	Ref	Core Contribution	Year	Field	Methodology & Purposes	Processing Steps		
						**Pre-processing**	**Processing**	**Post-processing**
49	[28]	MicrobiomeAnalyst 2.0	2023	Molecular and Cellular Omics	Implemented an R Shiny web framework using phyloseq, DESeq2, and PICRUSt2 for statistical, functional, and integrative analysis of microbiome data		※	※
50	[8]	ROB-MEN web-application	2023	General Tools	Integrated with CINeMA framework, semi-automated some of the required steps of the ROB-MEN tool and produced the two output tables in a ready-to-use .csv format			※
51	[18]	RetroBioCat	2023	Biochemistry	A python Flask web server that employs Jinja2 to render HTML pages, utilizing Bootstrap 4 and custom Javascript to provide the user interface, the Scikit-learn, Pandas, Biopython, and NumPy python packages were employed			※
52	[23]	Zero-shot Extraction	2023	Clinical Trials	Two models, GPT-3.5 Turbo and GPT-JT, were selected. Prompts were created through an iterative process with database curators, often including value lists for multiple-choice questions		※	
53	[55]	Chat2Brain	2023	Neuroscience	Used LLMs (ChatGPT) and a text-to-image model to map text queries to brain activation map	※		※
54	[25]	BUGSnet	2023	Social Science	Automated R package (Bayesian inference Using Gibbs Sampling)			※
55	[21]	Evaluate ChatGPT's performance	2024	Clinical Trials	Compare ChatGPT's performance in screening radiology abstracts with general physicians using sensitivity, specificity, PPV, NPV, and workload saving	※		
56	[29]	Evaluate the Efficacy of LLMs in MA	2024	General Tools	Evaluated efficacy of LLMs by comparing with expert reviews using statistical methods, analyzing metrics like accuracy, sensitivity, specificity, predictive values, F1-score, and Matthews correlation coefficient	※		
57	[41]	Evaluate LLMs in NMA	2024	Clinical Trials	Four case studies were used to develop a Python script that utilizes an LLMs to automate data extraction, NMA script generation, and report creation			※

Table continues on the next page...

No	Ref	Core Contribution	Year	Field	Methodology & Purposes	Processing Steps		
						Pre-processing	Processing	Post-processing
58	[45]	LLMs in data extraction of AMA	2024	Clinical Trials	Used the NCBI API to retrieve clinical trial papers, extracting key data from the XML content, and employing GPT to generate SQL queries for structuring and analyzing the data and saved as a CSV for further analysis		※	
59	[54]	MetaMate	2024	Social Science	Few-shot prompting for in-context learning		※	
60	[60]	Annotated a modest but granular evaluation dataset of RCTs	2024	Clinical Trials	Zero shot in LLMs		※	
61	[42]	metaGWASmanager	2024	Molecular and Cellular Omics	Based on R, Bash, and Python, involved customizing analysis scripts, prepared and validated phenotype and genotypic data, conducted GWAS, performed quality control, and finalized the meta-analysis using METAL.			※

References

[1] Gisina Alisa, Devyatkin Dmitry, Lukin Anton, Lupatov Alexey, Molodchenkov Alexey, and Kholodenko Irina. Method for biomedical information extraction of immunosuppressive cell properties. In *2021 IEEE Ural-Siberian Conference on Computational Technologies in Cognitive Science, Genomics and Biomedicine (CSGB)*, pages 210–213, 2021.

[2] A. Anisienia, R. M. Mueller, A. Kupfer, and T. Staake. Research method classification with deep transfer learning for semi-automatic meta-analysis of information systems papers. volume 2020-January, pages 6099–6108. IEEE Computer Society, 2021.

[3] Frank A. Bosco, Krista L. Uggerslev, and Piers Steel. MetaBUS as a vehicle for facilitating meta-analysis. *Human Resource Management Review*, 27(1):237–254, March 2017.

[4] A. A. Boyko, A. M. Kaidina, Y. C. Kim, A. Yu. Lupatov, A. I. Panov, R. E. Suvorov, and A. V. Shvets. A framework for automated meta-analysis: Dendritic cell therapy case study. In *2016 IEEE 8th International Conference on Intelligent Systems (IS)*, pages 160–166, 2016.

[5] P. L. Bremer, G. Wohlgemuth, and O. Fiehn. The BinDiscover database: a biology-focused meta-analysis tool for 156,000 GC–TOF MS metabolome samples. *Journal of Cheminformatics*, 2023.

[6] T. Burgard, M. Bosnjak, and R. Studtrucker. PsychOpen CAMA: Publication of community-augmented meta-analyses in psychology. *Res Synth Methods*, 13(1):134–143, January 2022.

[7] Lu Cheng, Dmitriy A. Katz-Rogozhnikov, Kush R. Varshney, and Ioana Baldini. Automated meta-analysis in medical research: A causal learning perspective. In *In ACM Conference on Health, Inference, and Learning*, April 2021.

[8] Virginia Chiocchia, Alexander Holloway, and Georgia Salanti. Semi-automated assessment of the risk of bias due to missing evidence in network meta-analysis: a guidance paper for the ROB-MEN web-application. *BMC Medical Research Methodology*, 23(1):223, October 2023.

[9] Hyungwon Choi, Ronglai Shen, Arul M Chinnaiyan, and Debashis Ghosh. A Latent Variable Approach for Meta-Analysis of Gene Expression Data from Multiple Microarray Experiments. *BMC Bioinformatics*, 8(1):364, December 2007.

[10] A. Craig, S. H. Bae, and C. Taswell. Bridging the semantic and lexical webs: Concept-validating and hypothesis-exploring ontologies for the Nexus-PORTAL-DOORS system. volume 2, pages 269–274. International Institute of Informatics and Systemics, IIIS, 2017.

[11] Thomas Pa Debray, Johanna Aag Damen, Richard D. Riley, Kym Snell, Johannes B. Reitsma, Lotty Hooft, Gary S. Collins, and Karel Gm Moons. A framework for meta-analysis of prediction model studies with binary and time-to-event outcomes. *Statistical Methods in Medical Research*, 28(9):2768–2786, September 2019.

[12] Z. Deng, K. Yin, Y. Bao, V. D. Armengol, C. Wang, A. Tiwari, R. Barzilay, G. Parmigiani, D. Braun, and K. S. Hughes. Validation of a Semiautomated Natural Language Processing-Based Procedure for Meta-Analysis of Cancer Susceptibility Gene Penetrance. *JCO Clin Cancer Inform*, 3, August 2019.

[13] D.A. Devyatkin, A.I. Molodchenkov, A.V. Lukin, Y.S. Kim, A.A. Boyko, P.A. Karalkin, J.-H. Chiang, G.D. Volkova, and A.Yu. Lupatov. Towards automated meta-analysis of biomedical texts in the field of cell-based immunotherapy. *Biomedical Chemistry: Research and Methods*, 2(3):e00109, 9 2019.

[14] Jérôme Dockès, Russell A Poldrack, Romain Primet, Hande Gözükan, Tal Yarkoni, Fabian Suchanek, Bertrand Thirion, and Gaël Varoquaux. NeuroQuery, comprehensive meta-analysis of human brain mapping. *eLife*, 9:e53385, March 2020.

[15] T. Donoghue and B. Voytek. Automated meta-analysis of the event-related potential (ERP) literature. *Sci Rep*, 12(1):1867, February 2022.

[16] Julia Feichtinger, Ramsay J. McFarlane, and Lee D. Larcombe. CancerMA: a web-based tool for automatic meta-analysis of public cancer microarray data. *Database*, 2012, January 2012.

[17] Julia Feichtinger, Ramsay J. McFarlane, and Lee D. Larcombe. Cancerest: a web-based tool for automatic meta-analysis of public est data. *Database*, 2014:bau024, 04 2014.

[18] W. Finnigan, M. Lubberink, L. J. Hepworth, J. Citoler, A. P. Mattey, G. J. Ford, J. Sangster, S. C. Cosgrove, B. Z. da Costa, R. S. Heath, T. W. Thorpe, Y. Yu, S. L. Flitsch, and N. J. Turner. RetroBioCat Database: A Platform for Collaborative Curation and Automated Meta-Analysis of Biocatalysis Data. *ACS Catal*, 13(17):11771–11780, September 2023.

[19] Fangxin Hong, Rainer Breitling, Connor W. McEntee, Ben S. Wittner, Jennifer L. Nemhauser, and Joanne Chory. RankProd: a bioconductor package for detecting differentially expressed genes in meta-analysis. *Bioinformatics*, 22(22):2825–2827, November 2006.

[20] Zicheng Hu, Chethan Jujjavarapu, Jacob J. Hughey, Sandra Andorf, Hao-Chih Lee, Pier Federico Gherardini, Matthew H. Spitzer, Cristel G. Thomas, John Campbell, Patrick Dunn, Jeff Wiser, Brian A. Kidd, Joel T. Dudley, Garry P. Nolan, Sanchita Bhattacharya, and Atul J.

Butte. MetaCyto: A Tool for Automated Meta-analysis of Mass and Flow Cytometry Data. *Cell Reports*, 24(5):1377–1388, July 2018.

[21] M. Issaiy, H. Ghanaati, S. Kolahi, M. Shakiba, A. H. Jalali, D. Zarei, S. Kazemian, M. A. Avanaki, and K. Firouznia. Methodological insights into ChatGPT's screening performance in systematic reviews. *BMC Medical Research Methodology*, 24(1), 2024.

[22] A. Kale, S. Lee, T. Goan, E. Tipton, and J. Hullman. Metaexplorer: Facilitating reasoning with epistemic uncertainty in meta-analysis. Hamburg, Germany, 2023.

[23] David Kartchner, Selvi Ramalingam, Irfan Al-Hussaini, Olivia Kronick, and Cassie Mitchell. Zero-shot information extraction for clinical meta-analysis using large language models. In Dina Demner-fushman, Sophia Ananiadou, and Kevin Cohen, editors, *The 22nd Workshop on Biomedical Natural Language Processing and BioNLP Shared Tasks*, pages 396–405, Toronto, Canada, July 2023. Association for Computational Linguistics.

[24] M. Lam, S. Awasthi, H. J. Watson, J. Goldstein, G. Panagiotaropoulou, V. Trubetskoy, R. Karlsson, O. Frei, C. C. Fan, W. De Witte, N. R. Mota, N. Mullins, K. Brügger, S. H. Lee, N. R. Wray, N. Skarabis, H. Huang, B. Neale, M. J. Daly, M. Mattheisen, R. Walters, and S. Ripke. RICOPILI: Rapid Imputation for COnsortias PIpeLIne. *Bioinformatics*, 36(3):930–933, February 2020.

[25] Y Liu, A Béliveau, Y Wei, and MY Chen. A gentle introduction to Bayesian network meta-analysis using an automated R package. *Multivariate Behavioral Research*, 2023.

[26] Maria Llambrich, Eudald Correig, Josep Gumà, Jesús Brezmes, and Raquel Cumeras. Amanida: an R package for meta-analysis of metabolomics non-integral data. *Bioinformatics*, 38(2):583–585, January 2022.

[27] M. W. Lorenz, N. A. Abdi, F. Scheckenbach, A. Pflug, A. Bülbül, A. L. Catapano, S. Agewall, M. Ezhov, M. L. Bots, S. Kiechl, A. Orth, G. D. Norata, J. P. Empana, H. J. Lin, S. McLachlan, L. Bokemark, K. Ronkainen, M. Amato, U. Schminke, S. R. Srinivasan, L. Lind, A. Kato, C. Dimitriadis, T. Przewlocki, S. Okazaki, C. D. A. Stehouwer, T. Lazarevic, P. Willeit, D. N. Yanez, H. Steinmetz, D. Sander, H. Poppert, M. Desvarieux, M. A. Ikram, S. Bevc, D. Staub, C. R. Sirtori, B. Iglseder, G. Engström, G. Tripepi, O. Beloqui, M. S. Lee, A. Friera, W. Xie, L. Grigore, M. Plichart, T. C. Su, C. Robertson, C. Schmidt, T. P. Tuomainen, F. Veglia, H. Völzke, G. Nijpels, A. Jovanovic, J. Willeit, R. L. Sacco, O. H. Franco, R. Hojs, H. Uthoff, B. Hedblad, H. W. Park, C. Suarez, D. Zhao, P. Ducimetiere, K. L. Chien, J. F. Price, G. Bergström, J. Kauhanen, E. Tremoli, M. Dörr, G. Berenson, A. Papagianni, A. Kablak-Ziembicka, K. Kitagawa, J. M. Dekker, R. Stolic, J. F. Polak, M. Sitzer, H. Bickel, T. Rundek, A. Hofman, R. Ekart, B. Frauchiger, S. Castelnuovo, M. Rosvall, C. Zoccali, M. F. Landecho, J. H. Bae, R. Gabriel, J. Liu, D. Baldassarre, and M. Kavousi. Automatic identification of variables in epidemiological datasets using logic regression. *BMC Med Inform Decis Mak*, 17(1), 2017.

[28] Y. Lu, G. Zhou, J. Ewald, Z. Pang, T. Shiri, and J. Xia. MicrobiomeAnalyst 2.0: Comprehensive statistical, functional and integrative analysis of microbiome data. *Nucleic Acids Research*, 51(1 W):W310–W318, 2023.

[29] Ronald Luo, Ziya Sastimoglu, Abu Ilius Faisal, and M. Jamal Deen. Evaluating the Efficacy of Large Language Models for Systematic Review and Meta-Analysis Screening, June 2024.

[30] Guillemette Marot, Jean-Louis Foulley, Claus-Dieter Mayer, and Florence Jaffrézic. Moderated effect size and P -value combinations for microarray meta-analyses. *Bioinformatics*, 25(20):2692–2699, October 2009.

[31] Matthew Michelson. Automating meta-analyses of randomized clinical trials: a first look. In *2014 AAAI Fall Symposium Series*, 2014.

[32] F. W. Mutinda, K. Liew, S. Yada, S. Wakamiya, and E. Aramaki. Automatic data extraction to support meta-analysis statistical analysis: a case study on breast cancer. *BMC Med Inform Decis Mak*, 22(1):158, June 2022.

[33] F. W. Mutinda, S. Yada, S. Wakamiya, and E. Aramaki. AUTOMETA: Automatic Meta-Analysis System Employing Natural Language Processing. *Studies in health technology and informatics*, 290:612–616, June 2022.

[34] Kishore Neppalli, Cornelia Caragea, Robin Mayes, Kim Nimon, and Fred Oswald. MetaSeer.STEM: Towards Automating Meta-Analyses. In *Proceedings of the AAAI Conference on Artificial Intelligence*, volume 30, pages 4035–4040, February 2016.

[35] Binod Neupane, Danielle Richer, Ashley Joel Bonner, Taddele Kibret, and Joseph Beyene. Network Meta-Analysis Using R: A Review of Currently Available Automated Packages. *PLOS ONE*, 9(12):e115065, December 2014.

[36] Gia H. Ngo, Minh Nguyen, Nancy F. Chen, and Mert R. Sabuncu. Text2Brain: Synthesis of Brain Activation Maps from Free-Form Text Query. *Medical Image Computing and Computer Assisted Intervention – MICCAI 2021*, 12907:605–614, 2021.

[37] Adriani Nikolakopoulou, Julian PT Higgins, Theodoros Papakonstantinou, Anna Chaimani, Cinzia Del Giovane, Matthias Egger, and Georgia Salanti. CINeMA: an approach for assessing confidence in the results of a network meta-analysis. *PLoS medicine*, 17(4):e1003082, 2020.

[38] Rhiannon K. Owen, Naomi Bradbury, Yiqiao Xin, Nicola Cooper, and Alex Sutton. MetaInsight: An interactive web-based tool for analyzing, interrogating, and visualizing network meta-analyses using R-shiny and netmeta. *Research Synthesis Methods*, 10(4):569–581, December 2019.

[39] Rafael Peñaloza. Towards a Logic of Meta-Analysis. In *Proceedings of the Seventeenth International Conference on Principles of Knowledge Representation and Reasoning*, pages 672–676, Rhodes, Greece, July 2020. International Joint Conferences on Artificial Intelligence Organization.

[40] Richeek Pradhan, David C. Hoaglin, Matthew Cornell, Weisong Liu, Victoria Wang, and Hong Yu. Automatic extraction of quantitative data from ClinicalTrials.gov to conduct meta-analyses. *Journal of Clinical Epidemiology*, 105:92–100, January 2019.

[41] T. Reason, E. Benbow, J. Langham, A. Gimblett, S. L. Klijn, and B. Malcolm. Artificial Intelligence to Automate Network Meta-Analyses: Four Case Studies to Evaluate the Potential Application of Large Language Models. *PharmacoEconomics - Open*, 8(2):205–220, 2024.

[42] Zulema Rodriguez-Hernandez, Mathias Gorski, Maria Tellez-Plaza, Pascal Schlosser, and Matthias Wuttke. metagwasmanager: a toolbox for an automated workflow from phenotypes to meta-analysis in gwas consortia. *Bioinformatics*, 40(5):btae294, 04 2024.

[43] S. Y. Ryu and G. A. Wendt. MetaMSD: meta analysis for mass spectrometry data. *PeerJ*, 2019.

[44] J. Sabates, S. Belleville, M. Castellani, T. Dwolatzky, B. M. Hampstead, A. Lampit, S. Simon, K. Anstey, B. Goodenough, S. Mancuso, D. Marques, R. Sinnott, and A. Bahar-Fuchs. CogTale: an online platform for the evaluation, synthesis, and dissemination of evidence from cognitive interventions studies. *Systematic Reviews*, 10(1), 2021.

[45] Fatemeh Shah-Mohammadi and Joseph Finkelstein. Large Language Model-Based Architecture for Automatic Outcome Data Extraction to Support Meta-Analysis. In *2024 IEEE 14th Annual Computing and Communication Workshop and Conference (CCWC)*, pages 0079–0085. IEEE, 2024.

[46] H. L. Shashirekha and Agaz Hussain Wani. Shinymde: Shiny tool for microarray meta-analysis for differentially expressed gene detection. In *2016 International Conference on Bioinformatics and Systems Biology (BSB)*, pages 1–5, 2016.

[47] Robert Suurmond, Henk Van Rhee, and Tony Hak. Introduction, comparison, and validation of *Meta-Essentials* : A free and simple tool for meta-analysis. *Research Synthesis Methods*, 8(4):537–553, December 2017.

[48] H. Thom, I. R. White, N. J. Welton, and G. Lu. Automated methods to test connectedness and quantify indirectness of evidence in network meta-analysis. *Research Synthesis Methods*, 10(1):113–124, 2019.

[49] G. Van Valkenhoef, S. Dias, A. E. Ades, and N. J. Welton. Automated generation of node-splitting models for assessment of inconsistency in network meta-analysis. *Research Synthesis Methods*, 7(1):80–93, 2016.

[50] Gert Van Valkenhoef, Guobing Lu, Bert De Brock, Hans Hillege, A. E. Ades, and Nicky J. Welton. Automating network meta-analysis. *Research Synthesis Methods*, 3(4):285–299, December 2012.

[51] Gert Van Valkenhoef, Tommi Tervonen, Tijs Zwinkels, Bert De Brock, and Hans Hillege. ADDIS: A decision support system for evidence-based medicine. *Decision Support Systems*, 55(2):459–475, May 2013.

[52] Wolfgang Viechtbauer. Conducting Meta-Analyses in *R* with the **metafor** Package. *Journal of Statistical Software*, 36(3), 2010.

[53] Xingbin Wang, Dongwan D. Kang, Kui Shen, Chi Song, Shuya Lu, Lun-Ching Chang, Serena G. Liao, Zhiguang Huo, Shaowu Tang, Ying Ding, Naftali Kaminski, Etienne Sibille, Yan Lin, Jia Li, and George C. Tseng. An R package suite for microarray meta-analysis in quality control, differentially expressed gene analysis and pathway enrichment detection. *Bioinformatics*, 28(19):2534–2536, October 2012.

[54] Xue Wang and Gaoxiang Luo. MetaMate: Large Language Model to the Rescue of Automated Data Extraction for Educational Systematic Reviews and Meta-analyses, 2024.

[55] Yaonai Wei, Tuo Zhang, Han Zhang, Tianyang Zhong, Lin Zhao, Zhengliang Liu, Chong Ma, Songyao Zhang, Muheng Shang, Lei Du, Xiao Li, Tianming Liu, and Junwei Han. Chat2brain: A method for mapping open-ended semantic queries to brain activation maps. In *2023 IEEE International Conference on Bioinformatics and Biomedicine (BIBM)*, pages 1523–1530, 2023.

[56] Cristen J. Willer, Yun Li, and Gonçalo R. Abecasis. METAL: fast and efficient meta-analysis of genomewide association scans. *Bioinformatics*, 26(17):2190–2191, September 2010.

[57] Zhaohan Xiong, Tong Liu, Gary Tse, Mengqi Gong, Patrick A. Gladding, Bruce H. Smaill, Martin K. Stiles, Anne M. Gillis, and Jichao Zhao. A Machine Learning Aided Systematic Review and Meta-Analysis of the Relative Risk of Atrial Fibrillation in Patients With Diabetes Mellitus. *Frontiers in Physiology*, 9:835, July 2018.

[58] Xu Yang, Haijing Tang, Xiaonan Dongye, and Guo Chen. Exploration of meta analysis automation. In *2018 International Conference on Network Infrastructure and Digital Content (IC-NIDC)*, pages 218–222, 2018.

[59] Tal Yarkoni, Russell A Poldrack, Thomas E Nichols, David C Van Essen, and Tor D Wager. Large-scale automated synthesis of human functional neuroimaging data. *Nature Methods*, 8(8):665–670, August 2011.

[60] Hye Sun Yun, David Pogrebitskiy, Iain James Marshall, and Byron C Wallace. Automatically extracting numerical results from randomized controlled trials with large language models. In Kaivalya Deshpande, Madalina Fiterau, Shalmali Joshi, Zachary Lipton, Rajesh Ranganath, and Iñigo Urteaga, editors, *Proceedings of the 9th Machine Learning for Healthcare Conference*, volume 252 of *Proceedings of Machine Learning Research*. PMLR, 8 2024.

[61] X. Zhang, C. Wang, Y. Yao, W. Sun, Y. Guo, L. Ma, X. Lu, and H. Deng. Construction of a Meta-Evidence Prototype Database of Traditional Chinese Medicine Splenogastric Diseases and Its Application in an Automatic Meta-Analysis System. *Evidence-Based Complementary and Alternative Medicine*, 2022:6933523, 2022.
